# Identification of ATF3 as a novel protective signature of quiescent colorectal tumor cells

**DOI:** 10.1038/s41419-023-06204-1

**Published:** 2023-10-13

**Authors:** Xi Lu, Lei Zhong, Emma Lindell, Margus Veanes, Jing Guo, Miao Zhao, Maede Salehi, Fredrik J. Swartling, Xingqi Chen, Tobias Sjöblom, Xiaonan Zhang

**Affiliations:** 1 Department of Immunology, Genetics and Pathology, Uppsala University, Uppsala, Sweden; 2 Department of Pharmacy, Personalized Drug Therapy Key Laboratory of Sichuan Province, Sichuan Provincial People’s Hospital, Sichuan, China; 3 Centre for Computational Biology, Duke-NUS Medical School, 8 College Road, 169857 Singapore, Singapore

**Keywords:** Cancer microenvironment, Targeted therapies, Chromatin structure

## Abstract

Colorectal cancer (CRC) is the third most common cancer and the second leading cause of death in the world. In most cases, drug resistance and tumor recurrence are ultimately inevitable. One obstacle is the presence of chemotherapy-insensitive quiescent cancer cells (QCCs). Identification of unique features of QCCs may facilitate the development of new targeted therapeutic strategies to eliminate tumor cells and thereby delay tumor recurrence. Here, using single-cell RNA sequencing, we classified proliferating and quiescent cancer cell populations in the human colorectal cancer spheroid model and identified ATF3 as a novel signature of QCCs that could support cells living in a metabolically restricted microenvironment. RNA velocity further showed a shift from the QCC group to the PCC group indicating the regenerative capacity of the QCCs. Our further results of epigenetic analysis, STING analysis, and evaluation of TCGA COAD datasets build a conclusion that ATF3 can interact with DDIT4 and TRIB3 at the transcriptional level. In addition, decreasing the expression level of ATF3 could enhance the efficacy of 5-FU on CRC MCTS models. In conclusion, ATF3 was identified as a novel marker of QCCs, and combining conventional drugs targeting PCCs with an option to target QCCs by reducing ATF3 expression levels may be a promising strategy for more efficient removal of tumor cells.

## Introduction

Colorectal cancer (CRC) is the third most common cancer but ranks second in mortality, with nearly 2 million incident cases and 935,000 deaths in 2020 [1]. Surgery followed by chemotherapy or radiotherapy is the mainstay of CRC treatment, and several targeted therapies for incurable metastatic CRCs have been developed [2]. Unfortunately, there is still a need for more effective treatments due to progressive drug resistance and, in most cases, tumor recurrence [3–6]. One obstacle is the existence of quiescent cancer cells (QCCs) that are in the nonproliferating G0 phase [7]. At present, most clinically used anticancer agents target proliferating cancer cells (S/G2/M, PCCs), making the phase of the cell cycle a major determinant of whether a cancer cell will respond to a given drug. However, more than 80% of internal cancer cells within a tumor have been reported to be quiescent, ultimately leading to the ineffective elimination of solid tumors [8]. The QCCs also induce nascent vessels in deeper regions of tumors after chemotherapy, which allows tumor regrowth after cessation of treatment [9], supporting the observation that tumors enriched in chemoresistant QCCs relapse significantly earlier than tumors harboring fewer QCCs under favorable conditions [10, 11]. Considering all the obstacles posed by QCCs in solid tumor treatments, innovative concepts to specifically target them in CRC are urgently needed.

Models that closely mimic the human cancer microenvironment and contain cancer cells in proliferating and quiescent states are important tools for understanding QCC-specific features and developing new therapeutic options to overcome QCC-related drug resistance [12]. Multi-cellular tumor spheroid (MCTS) is a 3D model that is heterogeneous with a well-defined geometry, containing proliferating cell populations at the surface layers with a positive expression of Ki67 and quiescent cancer cells in the core with a positive expression of p27Kip1 (cyclin-dependent kinase inhibitor) [13]. Furthermore, the size (volume) of each sphere is almost the same, making the results of subsequent experiments consistent and comparable. For this reason, MCTS is widely used in related studies and chemical screening [14–18].

In this study, we identified ATF3 as a novel hallmark in QCCs that could support cells living in a metabolically restricted microenvironment. Furthermore, ATAC-Seq data revealed that DDIT4 and TRIB3 have overall higher chromatin accessibility in cells treated with sangivamycin, leading to the discovery of the interaction of ATF3 with DDIT4 and TRIB3 at the transcriptional level. Further decreasing the expression level of ATF3 could enhance the efficacy of 5-FU on CRC MCTS models. It is expected that combining conventional drugs targeting PCC with options reducing ATF3 expression is expected to be effective in eradicating tumor cells and warrants further study.

## Materials and methods

### Chemicals and antibodies

Sangivamycin (HY-118384), rapamycin (HY-10219) and torin-1 (HY-13003) were obtained from MedChemExpress (NJ, USA). The primary antibodies used for western blotting were as follows: rabbit anti-ATF3 (#33593), rabbit anti-ATF4 (#118115) and rabbit anti-TRIB3 (#43043) were obtained from Cell signaling (MA, USA); rabbit anti-DDIT4 (#10638-1-AP) was obtained from Proteintech; mouse anti-actin (#sc-47778) was obtained from Santa Cruz Biotechnology (TX, USA). The ATF3 antibody (#18665) used for co-immunoprecipitation was obtained from Cell signaling (MA, USA). The primary antibodies used for immunochemistry staining: ki67 (#M7240, Agilent Technologies, CA, USA), p27 (#M72031, Agilent Technologies, CA, USA) and ATF3 (#MA5-31360, Thermo Scientific, MA, USA).

### Cell culture

HCT116 (#CCL-247), DLD-1 (#CCL-221), HT-29 (#HTB-38) and HEK293T (#CRL-3216) cells were obtained from ATCC (VA, USA). HCT116, DLD-1 and HT-29 CRC cells were maintained in McCoy’s 5A medium (#16600082, Thermo Scientific, MA, USA), HEK293T cells were maintained in DMEM medium (#10569010, Thermo Scientific, MA, USA) with the addition of 10% Fetal Bovine Serum (#10270106, Thermo Scientific, MA, USA) and 1% Penicillin-Streptomycin (#15140-122, Thermo Scientific, MA, USA) at 37 °C in 5% CO_2_. All cell lines were authenticated by STR profiling (ATCC cell authentication service) and regularly checked for mycoplasma infection with MycoAlert mycoplasma detection kit (InvivoGen, Toulouse, France).

### Generation of spheroids

For this, 10,000 cells/well were plated in 96-well ultra-low attachment plates (#7007, Corning, NY, USA) in 200 μL medium. Then plates were centrifuged at 1500 rpm for 10 min. For single-cell RNA-Seq assay, plates were incubated for 8d before spheroids trypsinization. For drug exposure, plates were incubated for 4d prior to the indicated treatments for cell viability assays or protein assays. The volume of spheroids was calculated by the equation Volume = (4/3)*3.14*R^3^ (R = radius).

### Single-cell RNA sequencing library preparation

Single-cell capture, lysis, reverse transcription, and pre-amplification were performed in the single-cell system following the manufacturer’s protocols by Eukaryotic Single-Cell Genomics Facility (Karolinska Institute, Sweden). Libraries were sequenced using the Illumina NovaSeq 6000 System.

### Single-cell RNA-Seq analysis

10x Genomics CellRanger 6.0.2 software [19] was used to demultiplex cell barcodes. Reads were mapped to the GRCh38 human reference transcriptome using the STAR aligner. The number of cells in samples was identified with a cell barcode in a library. Cells containing the number of counts <100 and the number of detected feature genes <100 and mitochondrial percentage >0.3 will be filtered. R package DoubletFinder [20] was used to distinguish between empty droplets and droplets containing a cell. The raw matrix of gene counts versus cells from CellRanger output was filtered, removing unqualified cells. R package Seurat^12^ was used to perform the downstream analysis of the matrix. Expression normalization was performed using the LogNormalize() function in Seurat. To adjust for difference in library size and cell cycle, the number of UMIs, mitochondrial content and cell-cycle difference were regressed using a linear model during gene scaling and centering. The uninteresting differences in the cell cycle could be removed using the “Alternate Workflow” in Seurat based on the expression level of previously published G2/M and S-phase gene signatures. Expression values were scaled across all the cells in a given dataset. Scaled z-score residuals (‘relative expression’) were used for dimensionality reduction and clustering. PCA was conducted on all expressed genes. Significant principal components were used as inputs for nonlinear dimensionality reduction techniques (UMAP) as well as cell clustering. Differential expression testing was performed using FindAllMarkers (Wilcoxon rank-sum test, FDR < 0.05) among clusters from corresponding resolutions. Genes with a detection rate difference between clusters of 0.1 or greater were included in differential testing. Hallmark pathway analysis was conducted using R package fgsea. Genes were assigned with AUC value using R package presto and then ranked to do the pathway enrichment. Pathways were selected based on FDR < 0.05.

### RNA velocity analysis

RNA velocities were calculated using scVelo [21] with default parameters, utilizing HCT cells derived from single-cell RNA sequencing (scRNA-seq) as input. The input cells underwent filtration and normalization through the *scvelo.pp.filter_and_normalize* function, with the parameter min_shared_counts set to 5. The top 30 principal components (PCs) were employed for moment computation in velocity estimation using the scvelo.pp.moments method. Terminal states and latent time were assessed through the scvelo.tl.latent_time function integrated within scVelo. To systematically identify putative driver genes responsible for transcriptional changes, high likelihoods in dynamic models were analyzed. The 300 highest-likelihood genes were selected and utilized for generating a heatmap via the scvelo.pl.heatmap function. These top-ranked genes were subsequently subjected to Gene Ontology analysis using the Database for Annotation, Visualization, and Integrated Discovery (DAVID) available at https://david.ncifcrf.gov/.

### RNA-Seq for ATF3 parental and KO MCTS

TRIzol (Life Technologies) was used for total RNA extraction from the frozen samples by following the manufacturer’s protocol. RNA was quantified by ultraviolet spectrophotometry, and RNA quality was assessed on the Agilent 2100 bioanalyser. RINs ranged from 6.3 to 9.1 (mean 8.2 ± 0.6). One microgram of total RNA was used to prepare the RNA-Seq libraries. RNA-Seq libraries were prepared with Illumina TruSeq RNA sample preparation kits by using the protocol for poly-A enriched mRNA. To avoid batch effects, samples were pooled (4–5 samples/pool, 2 lanes per pool). Finally, paired-end 2 × 100 bp sequencing was performed on the Illumina Hi-Seq platform (mean sequencing depth of 196 M). TopHat 2.0.12 with Bowtie2 2.2.3 and Samtools 0.1.18 was run by using human genome version GRch38 (hg38.78) reference genome. RNA-Seq read counts were computed with HTSeq 0.6.1. Counts are processed to cpm (counts per million), which is defined by dividing the mapped reads count by a per-million scaling factor of total mapped reads. Comparison of gene expression between conditions are ranked by log2 fold change. The functional interpretation of the differences between the groups are analyzed by Gene Set Enrichment test (GSEA) on the MSigDB hallmark gene sets using clusterProfiler (version 3.18.1) GSEA function with parameters minGSSize = 5, pvalueCutoff = 0.05.

### Western blot

For monolayer-based experiments, 700,000 cells were seeded into 60 mm dishes the day before treatment. For spheroid-based experiments, 10,000 cells/well were seeded in 96-well ultra-low attachment plates (#7007, Corning, NY, USA). After 4 days of growth, spheroids were treated for 24 or 72 h as indicated for protein assays. Then cells were harvested and lysed in ice-cold RIPA lysis buffer (#89901, Fisher Scientific) and run on 4–12% SDS-PAGE gels (#NP0336BOX, Thermo Scientific, MA, USA). Proteins were transferred to nitrocellulose membranes and incubated at room temperature in 5% non-fat dry milk in 1x PBST for 1 h. Membranes were then incubated overnight at 4 °C with primary antibodies: rabbit anti-ATF3 (1:1000), rabbit anti-ATF4 (1:1000), TRIB3 (1:1000), DDIT4 (1:1000) and mouse anti-actin (1:5000). Next day, membranes were washed with 1x PBST and incubated with secondary antibodies: anti-Rabbit (#31430, Thermo Scientific, MA, USA) or anti-Mouse (#31460, Thermo Scientific, MA, USA) at a dilution of 1:5000 for 1 h. Immunoreactive bands were detected by the Amersham Imager 680 system.

### Immunochemistry staining

Generated spheroids were fixated with 4% Formalin overnight and dehydrated with 70% EtOH for 2 h. The spheroids were then incorporated in 200 μL Histogel (#HG-4000-012, Fisher Scientific), embedded in paraffin and sectioned into 5 mm to glass slides using a microtome. After overnight incubation at 37 °C the slides were rehydrated and deparaffinized, 2*5 min in Xylene, 100%, 90% and 70% EtOH and H_2_O. Antigens were retrieved in 1:100 antigen unmasking solution (#H-3300, Vector Laboratories Inc, CA, USA) microwaved at max effect until boiling point and then at 100 W for 15 min. After cooling down for ~30 min the slides were washed 3*4 min in TBS-T buffer (Sigma-Aldrich, #91414-100TAB) before blocking in a humidity chamber (Simport Scientific, Canada, # 631-1923) with 2.5% Normal Horse Serum (Vector Laboratories Inc, Cat #S-2012) for 25 min. Spheroids for Hematoxylin staining were washed 3*4 min with TBS-T before staining with Hematoxylin (1:10, Agilent Technologies, Inc, #S3309) for ~20 s and washed in H_2_O before mounting (Agilent Technologies Inc, #S3025). Additional spheroids were stained with primary antibodies ki67 (1:200, Agilent Technologies, CA, USA, #M7240), p27 (1:200, Agilent Technologies Inc, #M7203) and for HEK293T 1:100 and HCT116 1:50 with ATF3 (#MA5-31360, Thermo Fisher Scientific, MA, USA) in 2.5% Normal Horse serum, overnight in the humidity chamber. The slides were washed 3*5 min in TBST and counterstained applying ImPRESS (Peroxidase) Polymer Anti-Mouse IgG Reagent (Vector Laboratories Inc, #MP-7402) for 15 min in humidity chamber. After the second staining, the spheroids were washed in TBST 3*4 min before the antigen was developed, applying DAB Substrate Kit (Vector Laboratories Inc, #SK-4100) until staining occurred. The slides were mounted after being washed in TBST and H_2_O.

### Immunoprecipitation analysis

Cells were seeded onto 10 cm dishes and treated with 100 nM sangivamycin when the cells reached 70% confluency. Cells were harvested 24 h after sangivamycin treatment. Subsequent immunoprecipitation assays were performed as described in the Dynabeads® (#14321D, Thermo Fisher Scientific) co-immunoprecipitation protocol.

### Generation of CRISPR/Cas9-mediated ATF3 knockout (KO) cells

HCT116 ATF3 KO pool cells were generated using CRISPR/Cas9 by Synthego (CA, USA) with the sgRNA sequence: AGAAGGCACUCACUUUCUGC. PCR and Sequencing Primers: F: TTTCGGGGTCTTTTAGCGCT, R: TGCTTTGCCACCTCCTCATT. Approximately 200 single cells per well were prepared. After 1 week, ~50 single cells that could expand into colonies were expanded in 12-well plates. Next, ~15 colonies were further expanded in 6-well plates and tested by western blotting. Finally, two clones (KO1 and KO2) were selected.

### Cell viability assay

Cell viability assay was performed using resazurin-based cell viability assay where cells were plated in a 96-well plate at a density of 10,000 cells/well. Plated cells were then incubated for 24 h before exposure to treatment for 72 h at 37 °C. Monolayer cells and MCTS were incubated with resazurin (#R7017, Sigma-Aldrich) solution for 2.5 h and overnight, respectively, at 37 °C and the fluorescent intensity was read using CLARIOstar microplate reader (545-20/600-40 nm) filter set. The percentage of cell viability was calculated as (fluorescence intensity of treated cells − fluorescence intensity of background) / (fluorescence intensity of DMSO treated control − fluorescence intensity of background) × 100%. The half-maximal inhibitory concentration (IC_50_) was calculated using nonlinear regression, [Inhibitor] vs. normalized response analysis, in GraphPad prism 9.

### Drug screening

The screening was performed in 96w format using Mechanistic screening set at a concentration of 1 mM (Library description: Mechanistic Set VI, https://dtp.cancer.gov). The Mechanistic Set VI, which consists of 811 compounds, was derived from the 37,836 open compounds that have been tested in the NCI human tumor 60 cell line screen. A set of 324 compounds with >20% cytotoxicity at 10 μM toward HCT116 monolayer cells were progressed to HCT116 ATF3^parental^ MCTS-based selection, where 93 compounds with >50% cytotoxicity at 10 μM toward HCT116 ATF3^parental^ MCTS were selected. The 93 hits were then further tested against ATF3^parental^ *and* ATF3^KO^ *cells in 2D conditions*, 10 compounds were selected (NSC143648, 328587, 123115, 102815, 166454, 241906, 320864, 651079, 177365 and 359463) using selection criteria: IC_50_ (ATF3^Par^/ ATF3^KO^) $\ge$2. 10 hits were further tested on scramble and siATF3 HT-29 cells, NSC 328587, 177365 and 359463 were finally selected as hit compounds using selection criteria: IC_50_ (ATF3^Par^/ ATF3^KO^) $\ge$2.

### Bulk ATAC-Seq sequencing library preparation

Cells treated with 100 nM sangivamycin for 24 h were fixed with 1% formaldehyde (Thermo Fisher, #28906) for 10 min and quenched with 0.125 M glycine for 5 min at room temperature. After the fixation, ATAC-Seq was performed as previously described [22]. Cells were counted and 50.000 cells were used per ATAC-Seq reaction. The transposition reaction followed the normal ATAC-Seq protocol. After transposition, a reverse crosslink solution (50 mM Tris-Cl PH 8.0 (Invitrogen, #15568-025), 1 mM EDTA (Invitrogen, #AM9290G), 1% SDS (Invitrogen, #15553-035), 0.2 M NaCl (Invitrogen, #AM9759) and 5 ng/µL proteinase K (Thermo Scientific, #EO0491)) was added up to 200 µL. The mixture was incubated at 65 °C with 1200 rpm shaking in a heat block overnight, then purified with MinElute PCR Purification kit (QIAGEN, #28004) and eluted in 10 µL Qiagen elution buffer. Sequencing libraries were prepared using the original ATAC-Seq protocol [23]. The sequencing was performed on Illumina NovaSeq 6000, and at least 20 million paired-end sequencing reads were generated for each ATAC-Seq library.

### Bulk ATAC-Seq analysis

The adapter sequences were trimmed first and then the ATAC-Seq sequencing reads were mapped to genome hg38 using bowtie2 [24]. Mapped paired reads were corrected for the Tn5 cleavage position with shifting +4/−5 bp depending on the strand of reads. All mapped reads were extended to 50 bp centered by Tn5 offset. The PCR duplication was removed using Picard and reads mapping on chromosome M were removed. The peak-calling process of each ATAC-Seq library was performed with MASC2 [25] with parameters -f BED, -g hs, -q 0.01, --nomodel, --shift 0. Detected peaks were merged into a raw matrix with bedtools [26]. Raw reads within peaks were normalized using EdgeR’s cpm [27]. Log transformation was applied on the normalized peaks to calculate the Pearson correlation among duplicates. Differential ATAC peaks for sangivamycin were selected using R package DESeq2 [28], with cutoff : FDR < 0.05, |log2 fold change| > 1 and peak average intensity >20. Software HOMER [29] was used to identify motif binding sites. Peak visualization was done using the software IGV [30].

### Synergy effect analysis

Cells were treated with indicated concentrations of 5-FU, rapamycin, or torin-1 for 72 h, and viability was determined using a resazurin-based assay. The coefficient of drug interaction (CDI) was used to analyze the interactions between rapamycin/torin-1 and 5-FU. According to CDI values, the interactions were categorized as synergistic, additive or antagonistic, respectively. CDI was calculated as follows: CDI = AB/(A × B) where: AB = cell viability value for the combination of rapamycin/torin-1 and 5-FU; A and B = cell viability value for the single treatment of rapamycin/torin-1 or 5-FU. A CDI value <1, =1 or >1 indicates that the drugs are synergistic, additive, or antagonistic, respectively. A CDI value less than 0.7 indicates that the drugs are significantly synergistic [31].

### Statistical analysis

The data was expressed as the mean ± SD (*n* = 3) of three independent experiments. IC_50_ was calculated using nonlinear regression, [Inhibitor] vs. normalized response analysis, GraphPad prism 9. Asterisks denote significant, * *p* < 0.05; ** *p* < 0.01; *** *p* < 0.001; **** *p* < 0.0001.

## Results

### Single-cell analysis identifies PCC and QCC populations

**Fig. 1:**
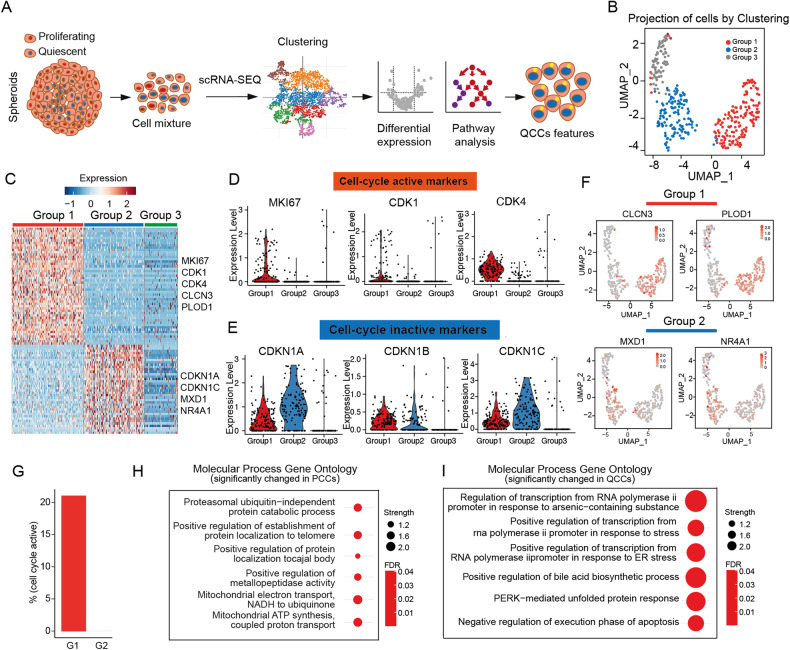
PCC and QCC populations were separated by single-cell RNA-Seq. **A** Graphical overview of identification of PCCs and QCCs by single-cell RNA-Seq. **B** UMAP visualization of MCTS model by the presence of three cell clusters. **C** Heatmap of the expression of the most characterized genes for each cluster. **D** Bar plot of gene expression for cell-cycle active markers. **E** Bar plot of gene expression for cell-cycle inactive markers. **F** UMAP visualization of representative expression genes of Group1 and Group2. **G** Percentage of cell-cycle active cells in Group1 and Group2. **H** Top 6 significant enriched GO terms in PCCs (Group1). **I** Top 6 significant enriched GO terms in QCCs (Group2).

In our previous studies, we established HCT116 and HT-29 CRC MCTS models which could mimic the real conditions in solid tumors, containing PCCs (p27^low^ and Ki67^high^) on the rim and QCCs (p27^high^ and Ki67^low^) in the core [13, 32–34]. To further understand the features of two groups, we isolated HCT116 MCTS into single cells and performed single-cell RNA-Seq (Fig. 1A). The correlation between number of counts per cell and the number of detected genes per cell was 0.88 (Supplementary Fig. S1A), and the percent of mitochondria per cell was below 25% (Supplementary Fig. S1B), indicating a good quality of cells used for downstream analysis. Unsupervised two-dimensional uniform manifold approximation and projection (UMAP) revealed three distinct clusters (Fig. 1B). We identified 1855 unique genes for PCCs and 130 unique genes for QCCs (FDR < 0.05). We noticed that Group 1 exhibits high expression levels of MKI67, CDK1, CDK4, CLCN3 and PLOD, and Group 2 exhibits high expression levels of CDKN1A, CDKN1C, MXD1 and NR4A1 (Fig. 1C). MKI67, CDK1 and CDK4 are positive cell-cycle regulators, and CDKN1A and CDKN1C are negative cell-cycle regulators [35]. However, Group 3 with low cell number did not exhibit any notable features regarding cell-cycle markers (high expression level of proliferating markers MKI67, CDK1 and CDK4, and high expression level of quiescent markers CDKN1A, CDKN1C) (Fig. 1D, E; see Supplementary Table S1 for a full list of marked genes into different groups). We also looked into the distribution of annotated genes CLCN3, PLOD1, MXD1 and NR4A1 in three clusters. Chloride channel protein 3 (CLCN3) and procollagen-lysine, 2-oxoglutarate 5-dioxygenase 1 (PLOD1), with a specific allocation to Group 1, were reported to be involved in cancer cell proliferation and tumor progression [36, 37], whereas MXD1 and NR4A1 are allocated to Group 2 (Fig. 1F). MYC-associated factor X dimerization transcription factor 1 (MXD1), a MYC antagonist, is a tumor suppressor and opposes the functions of oncogene MYC [38, 39]. Nuclear receptor subfamily 4A1 (NR4A1) plays diverse roles in different tumors. NR4A1 overexpression may attenuate malignant progression in some tumors and may also serve as a drug target for cancer chemotherapy [40, 41].

Next, we calculated the percentage of the cell-cycle active population, where Group 1 contained 20% of the cell-cycle active population, but almost zero in Group 2 (Fig. 1G). Taken together, we concluded Group 1 as the proliferating cancer cells (PCCs) and Group 2 as the quiescent cancer cells (QCCs). Additionally, we noticed that the PCC group revealed significant changes in molecular pathways of mitochondrial electron transport, mitochondrial ATP synthesis, and proteasome ubiquitin-independent protein catabolic processes (Fig. 1H, a full list of altered GO pathways in PCC see Supplementary Table S2), consistent with the features described for cell-cycle active cells [42, 43]. Interestingly, the most pronounced changes in the QCC population tended to be related to RNA polymerase II regulation and response to stress (Fig. 1I, a full list of altered GO pathways in QCC, see Supplementary Table S3).

### Identification of ATF3 as a signature of QCCs

**Fig. 2:**
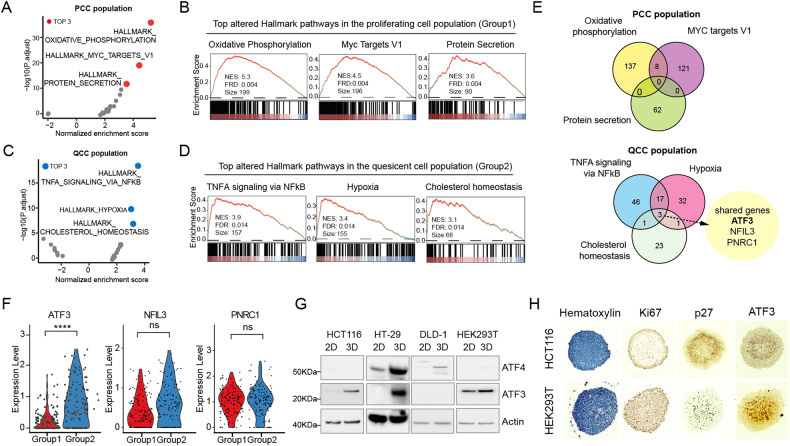
Grouped PCC and QCC populations exhibited distinct features and ATF3 is a hallmark of QCCs. **A** Scatterplot for hallmark pathways in the PCC population (Red dots indicate the top 3 significantly changed pathways). **B** GSEA enrichment profile of the top 3 altered hallmark pathways in the proliferated cell population, including Oxidative phosphorylation, MYC targets-V1, and Protein secretion. **C** Scatterplot for hallmark pathways in the QCC population (blue dots indicate the top 3 significantly changed pathways). **D** GSEA enrichment profile of the top 3 altered hallmark pathways in the quiescent cell population, including TNFα signaling via NF-kB, Hypoxia, and Cholesterol homeostasis. **E** Venn plot for overlapping genes involved in corresponding hallmark pathways in PCC and QCC populations. In the QCC population, ATF3, NFIL3 and PNRC1 are co-shared by the corresponding pathways: TNFα signaling via NF-kB, Hypoxia, and Cholesterol homeostasis. **F** Violin plots of gene expression of ATF3, NFIL3, and PNRC1 in Group 1 (PCCs) and Group 2 (QCCs) (****, *p* < 0.0001). **G** Expression levels of ATF3 and ATF4 in HCT116, HT-29, DLD-1 and HEK293T cells under both monolayer and MCTS conditions. **H** Immunochemistry staining for Ki67, p27 and ATF3 on HCT116 and HEK293T MCTS.

Since most anticancer agents used in the clinic are designed to target proliferating cells (S/G2/M), we aimed to discover novel vulnerabilities in the quiescent cancer cell population. Gene set enrichment analysis (GSEA) revealed distinct alterations that, in PCCs, oxidative phosphorylation, MYC targets_V1 and protein secretion were characterized as the most prominently enriched hallmark pathways (Fig. 2A, B, a full list of altered hallmark pathways in PCCs see Supplementary Table S4), and TNFA signaling via NFkB, hypoxia and cholesterol homeostasis were the most significantly upregulated pathways in QCCs (Fig. 2C, D, a full list of altered hallmark pathways in QCCs see Supplementary Table S5). We are curious which type of genes are enriched in corresponding hallmark pathways in PCCs and QCCs. To our surprise, the top three pathways in QCCs shared three genes (ATF3, NFIL3 and PNRC1) (Fig. 2E and Supplementary Fig. S2A). We further compared the expression levels of ATF3, NFIL3 and PNRC1 in PCCs and QCCs groups, and ATF3 showed a significant difference between two groups but not NFIL3 and PNRC1 (Fig. 2F). ATF3 (activating transcription factor 3) is a stress-induced transcription factor [44, 45], and has been reported to be induced by low glucose [46] and endoplasmic reticulum (ER) stress [47] which are featured microenvironment of QCCs [48, 49]. To ensure that high expression of ATF3 is a common feature of the QCC population, we further investigated ATF3 expression levels in several monolayer and MCTS systems (HCT116, HT-29, DLD-1 and HEK293T, which could form neat spheroids, Supplementary Fig. S2B). A consistent pattern of higher ATF3 expression was noted in HCT116, HT-29 and HEK293T MCTS conditions. In DLD-1 MCTS, instead of ATF3, the expression level of ATF4, which has been shown to tightly interact with ATF3 and is required in ATF3-induced responses [50], was higher than that of monolayer (Fig. 2G). In addition, we stained two different MCTS models and observed that Ki67 is mainly located at the edge of MCTS, p27 and ATF3 are mainly located in the core of MCTS (Fig. 2H). To further investigate the possibility of ATF3 as a signature of QCCs at the clinical level, we accessed the public dataset (GSE146771) and analyzed the provided scRNA-Seq data from 10 patient samples [51], following the same strategy performed for our previous data (Fig. 1). UMAP revealed eight distinct clusters, two CD45 positive immune cell populations and five vimentin positive populations whose expression is more correlated with the invasive phenotype of gastric cancer [52] (Supplementary Fig. S2C). We further investigated the expression of cell-cycle active markers (MKI67, CDK1, and CDK4), cell-cycle inactive markers (CDKN1A, CDKN1B, and CDKN1C), novel QCC marker ATF3, and ATF4 in the five groups. Interestingly, Group 7 had higher expression levels of cell-cycle active markers but not ATF3, whereas Group 5 showed higher expression levels of cell-cycle inactive marker and ATF3, but not MKI67, supporting our MCTS scRNA-Seq result that ATF3 is a QCC-specific marker (Supplementary Fig. S2D). ATF4 is expressed in groups expressing either CDKN1B or MKI67, indicating that ATF4 is not a QCC-specific marker in clinic (Supplementary Fig. S2D). Taken together, we concluded that high expression of ATF3 is a signature of QCCs.

### QCCs exhibit an extrapolated future proliferation state

**Fig. 3:**
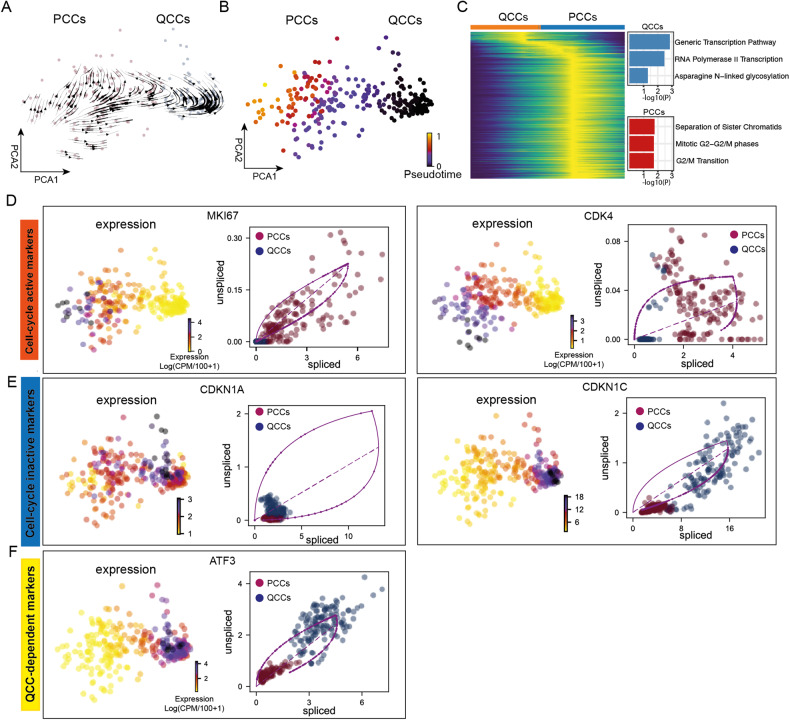
QCCs could repopulate into PCCs. **A** Dynamic streams predicted by RNA velocity analysis with a dynamical model. Arrows indicate the direction of the change in cell status based on the mRNA maturation in QCC and PCC groups. **B** Pseudotime predicted by the same workflow in (**A**). **C** A heatmap representation of the pseudotime inference shown in (**A**) and (**B**) in QCC and PCC groups. **D** Gene expression and phase portrait of cell-cycle activity markers MKI67 and CDK4. **E** Gene expression and splicing rate of cell-cycle inactive markers CDKN1A and CDKN1C. **F** Gene expression and splicing rate of QCCs-dependent markers ATF3.

To further understand the models of cell dynamics in both PCCs and QCCs groups, we performed RNA velocity analysis, which enables us to estimate dynamic time scales of the gene expression state by analyzing spliced and unspliced mRNA [53]. Interestingly, the RNA velocity showed transitions in the extrapolated future state between QCCs and PCCs, suggesting a dynamic process between these two groups (Fig. 3A). Further analysis showed that the pseudotime gradually increases from QCCs to PCCs (Fig. 3B–C, Supplementary Fig. S3A, and Supplementary Table S6), indicating that QCCs are able to repopulate into PCCs. We also tried to look into the connection of Group 3, which did not exhibit any notable features regarding cell-cycle markers. Interestingly, the estimated dynamic time scale indicated that Group 3 starts at the earliest pseudotime, where groups 1, 2 and 3 co-exist (Supplementary Fig. S3B). The gene ontology terms analysis revealed that Group 3 was enriched in biological functions pertaining to cytoplasmic translation, maintenance of location in cell and regulation of generation of precursor metabolites and energy pathway (Supplementary Fig. S3C). Next, we specifically investigated two cell-cycle activity markers, MKI67 and CDK4, both are induced and highly expressed in PCCs (Fig. 3D and Supplementary Fig. S3D). Cell-cycle arrest markers, CDKN1A and CDKN1C, as well as ATF3, showed higher expression levels in QCCs (Fig. 3E, F and Supplementary Fig. S3D).

### Cells lacking ATF3 show upregulated mitochondrial activity and are more sensitive to anticancer agents

**Fig. 4:**
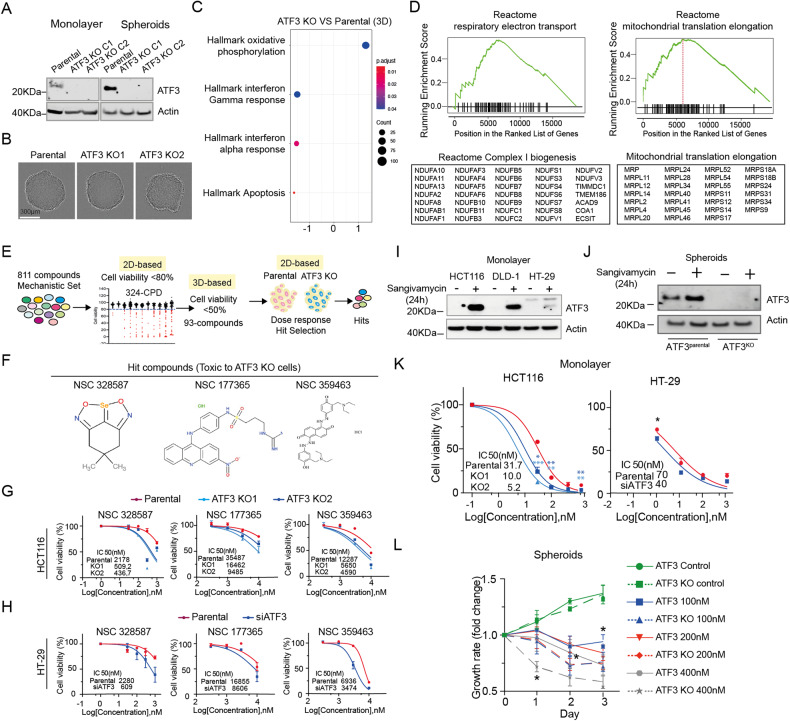
Cells lacking ATF3 show upregulated mitochondrial function and are more sensitive to anticancer agents. **A** Expression levels of ATF3 in ATF3^parental^ and ATF3^KO^ cells under monolayer and MCTS conditions. Actin is the loading control. Three independent experiments were performed. **B** Images of ATF3^parental^ and ATF3^KO^ MCTS taken by Incucyte, Scale bar: 300 µm. **C** GSEA enrichment profile of the top-altered hallmark pathways in HCT116 MCTS lacking ATF3 compared with HCT116 ATF3^parental^ MCTS. **D** Reactome analysis showing upregulation of the respiratory electron transport pathway and mitochondrial translation elongation in ATF3-deficient HCT116 MCTS. Table lists genes involved in complex I biogenesis and mitochondrial translation elongation. **E** Flow chart of hit compound identification using mechanistic screening set (Library description: Mechanistic Set VI, https://dtp.cancer.gov). **F** NSC numbers and chemical structures of the hit compounds. **G** Dose response to hit compounds in HCT116 ATF3^parental^ and ATF3^KO^ cells under monolayer conditions. The half-maximal inhibitory concentration (IC_50_) was calculated using nonlinear regression, [Inhibitor] vs. normalized response analysis. **H** Dose response to hit compounds in HT-29 ATF3^scramble^ and ATF3^KD^ cells under monolayer conditions. The half-maximal inhibitory concentration (IC_50_) was calculated using nonlinear regression, [Inhibitor] vs. normalized response analysis. **I** Expression levels of ATF3 in HCT116, DLD-1 and HT-29 cells treated with DMSO or 100 nM sangivamycin for 24 h under monolayer conditions. Actin is the loading control. **J** Expression levels of ATF3 in ATF3^parental^ and ATF3^KO^ cells treated with DMSO or 100 nM sangivamycin for 24 h under MCTS conditions. Actin is the loading control. **K** Dose response to sangivamycin. HCT116 ATF3^parental^ and ATF3^KO^ cells were treated with sangivamycin in monolayer condition for 72 h. The data is expressed as the mean ± SD (*n* = 3, unpaired *t*-test, two-stage step-up (Benjamini, Krieger, and Yekutieli); * *p* < 0.05, ** *p* < 0.01, *** *p* < 0.001). The half-maximal inhibitory concentration (IC50) was calculated using nonlinear regression, [Inhibitor] vs. normalized response analysis. **L** MCTS treated with indicated concentrations of sangivamycin was monitored in Incucyte for 3 days. Images for each spheroid were taken every day. The volume of spheroids was calculated by equation Volume = (4/3)*3.14*R^3^ (R = radius). The data is expressed as the mean ± SD (*n* = 3, using multiple-unpaired *t*-test, * *p* < 0.05).

To examine the role of ATF3 in QCCs, we first established ATF3 knockout cells in HCT116 using CRISPR-Cas9 editing and compared ATF3 expression levels in monolayer and MCTS conditions. Consistent with previous results (Fig. 2G), ATF3 protein levels were higher in MCTS compared to monolayers in ATF3^parental^ cells and were abolished in ATF3^KO^ clones (Fig. 4A). Compared to ATF3^parental^ MCTS, there is no noticeable shape change in ATF3^KO^ MCTS (Fig. 4B). We further compared the growth rates between ATF3^parental^ and ATF3^KO^ clones under monolayer and MCTS conditions, and there was no significant growth change between ATF3^parental^ and ATF3^KO^ clone 1 and clone 2 over 72 h in monolayer and 7d in MCTS conditions (Supplementary Fig. S4A). Next, we performed RNA-Seq on ATF3^parental^ and ATF3^KO^ MCTS and compared their expression pattern by gene set enrichment analysis. Our data indicated that the hallmark oxidative phosphorylation pathway was upregulated in ATF3^KO^ MCTS (Fig. 4C), consistent with the finding from Yin et al. [54]. Further reactome pathway analysis revealed upregulation of complex I biogenesis and mitochondrial translation elongation in cells lacking ATF3 (Fig. 4D, a full list see Supplementary Table S7). This result supports our finding that OXPHOS is enriched in PCCs where the expression level of ATF3 is lower compared with QCCs (Fig. 1H).

To discover ATF3-dependent therapeutic options for quiescent cancer cells, we further initiated a chemical screen using the mechanistic set provided by the National Institutes of Health (NCI), hoping to find a compound targeting cells expressing ATF3 (Fig. 4E). 811 compounds, derived from the 37,836 open compounds, were screened at both monolayer and spheroid models and 3 compounds (NSC328587, NSC177365 and NSC359463, Fig. 4F) showed a consistent specific toxicity to HCT116 ATF3^KO^ cells (Fig. 4G) and HT-29 ATF3^KD^ cells (Fig. 4H and Supplementary Fig. S4B), however, we did not identify any compounds targeting ATF3^parental^ cells, suggesting that cells lacking ATF3 are more sensitive to anticancer agents. To further illustrate the role of ATF3 in CRC, we treated cells with sangivamycin (Supplementary Fig. S4C), which was reported to upregulate the expression level of ATF3 [55] and inhibit the proliferation of a variety of human cancer cells, including colon carcinoma cells [56], a significant increase in ATF3 expression was observed in sangivamycin-treated samples under both monolayer (Fig. 4I) and MCTS conditions but not in ATF3^KO^ cells (Fig. 4J). Next, we compared the sensitivity of cells present or absent ATF3 to sangivamycin after 72 h exposure. IC_50_ in ATF3^parental^ is triple that in ATF3^KO^ HCT116 cells and IC_50_ in ATF3^scrable^ is almost double that in ATF3^KD^ HT29 cells (Fig. 4K). We also compared the volume of spheroids generated from ATF3^parental^ and ATF3^KO^ cells treated with the indicated concentrations of sangivamycin and noted a greater reduction in volume in ATF3^KO^ spheroids (Fig. 4L). Taken together, we concluded that CRC cells with the ability to upregulate ATF3 exhibit a stronger tolerance to anticancer reagents.

### ATF3 interacts with DDIT4 and TRIB3 at the transcriptional level

**Fig. 5:**
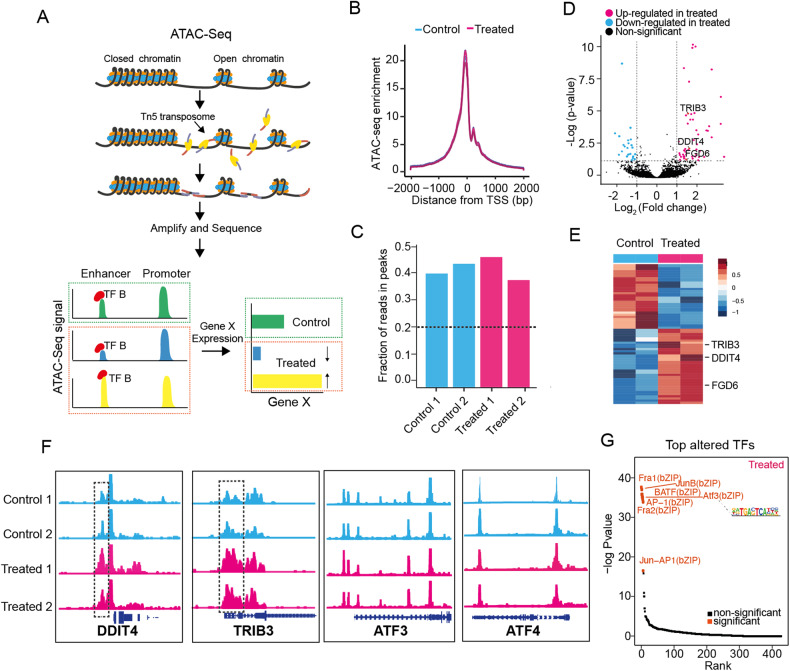
ATF3 could interact with TRIB3 and DDIT4 at the chromatin level. **A** Schematic illustration of the overall experimental designs. ATAC-Seq stands for Assay for Transposase-Accessible Chromatin with high-throughput sequencing. The ATAC-Seq method relies on next-generation sequencing (NGS) library construction using the hyperactive transposase Tn5. NGS adapters are loaded onto the transposase, which allows simultaneous fragmentation of chromatin and integration of those adapters into open chromatin regions. The libraries generated from cells treated with DMSO or 100 nM sangivamycin for 24 h were sequenced by NGS, and the regions of the genome with open or accessible chromatin are analyzed using bioinformatics. **B** Enrichment score of ATAC-Seq samples at transcription start sites (TSS) from treated and untreated cells. **C** Fraction of reads in peaks (FRiP) in duplicate samples. The dashed line shows the minimum FRiP score required for further analysis of that sample. **D** Volcano plot for enrichment transcription factors in the sangivamycin (100 nM, 24 h) treated group. **E** Heatmap of unique accessible chromatin regions of two groups treated with or without sangivamycin (100 nM, 24 h). Representative genes annotated to the chromatin regions are indicated. **F** Genome browser tracks of ATAC-Seq data for each sample of ATF3, ATF4, TRIB3 and DDIT4. Tracks show significant peak signal intensity (*y*-axis) for open chromatin regions of indicated genes (TRIB3 and DDIT4) (*x*-axis) in treated groups (pink, bottom) compared to the control groups (blue, top). Individual gene maps are shown in dark blue. **G** Scatter plot for enrichment transcription factors in the Sangivamycin group. ATF3 was one of the top-altered TFs.

ATF3 regulates chromatin accessibility and is responsible for the establishment and maintenance of open chromatin orchestration with gene expression in multiple cell types [57, 58], allowing us to speculate that the effect of sangivamycin on the expression of ATF3 may be partly related to remodeling of chromatin accessibility. To address this possibility, we decided to perform ATAC-Seq to examine the changes in chromatin accessibility before and after the treatment of sangivamycin (Fig. 5A). The ATAC-Seq data from all samples were determined to be of high quality based on analyses of enrichment score at transcription start sites (TSS) (Fig. 5B), fraction of reads in peaks (FRiP) (Fig. 5C), reproducibility among the technical replicates (Supplementary Fig. S5A) and distance matrix (Supplementary Fig. S5B). To delineate the chromatin-accessible difference between cells treated with or without sangivamycin, we applied the pairwise comparison and discovered totally 65 differential peaks across the whole genome, including 21 from untreated cells and 44 from treated cells (Fig. 5D, a full list see Supplementary Table S8). We further looked into the differential chromatin open regions in sangivamycin-treated samples, peaks were annotated to nearby genes *TRIB3*, *DDIT4* and *FGD6* (Fig. 5E). ATAC-Seq genome tracks showed a significant change in chromatin accessibility at the promotor regions of genes *TRIB3*, *DDIT4* and *FGD6*, while no significant change was annotated in chromatin open regions of *ATF3* and *ATF4* (Fig. 5F, Supplementary Fig. S5C). Motif discovery analysis revealed motif enrichment in treatment-specific ATAC peaks, with the transcription factor ATF3 topping in the rank (*p* < 0.01) (Fig. 5G). Meanwhile, a ChIP-seq dataset from the ENCODE Transcription Factor Targets dataset revealed that the ATF3 transcription factor targets 8397 genes, including DDIT4 and TRIB3. Taken together, we concluded that ATF3 likely interacts with *DDIT4* and *TRIB3* at the transcriptional level, thus altering their chromatin accessibility.

### ATF3/4, DDIT4 and TRIB3 are interrelated at the translational level

**Fig. 6:**
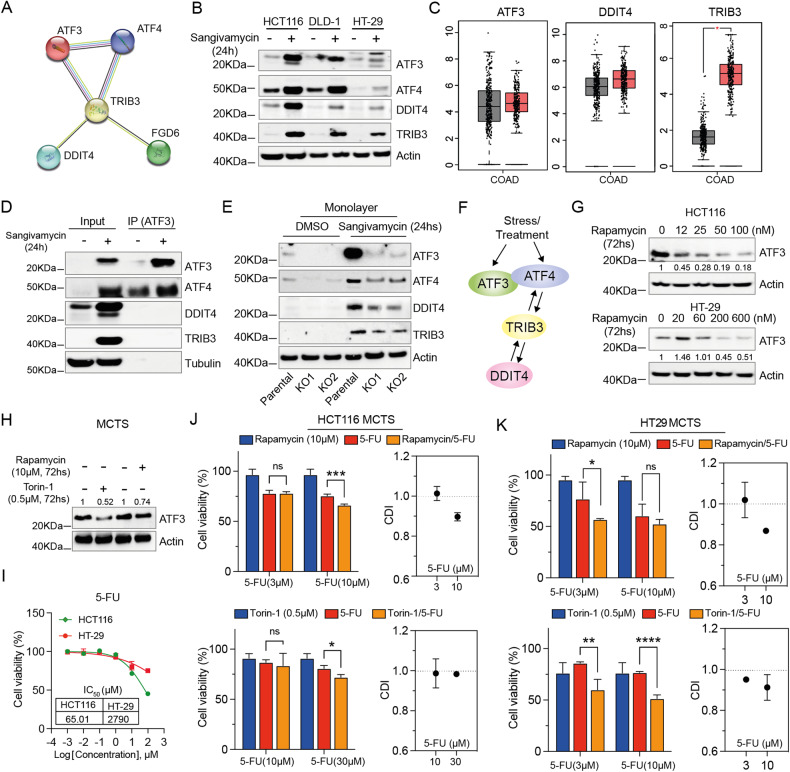
Lowering ATF3 improves the effect of 5-FU on CRC MCTS. **A** Plot for protein interactions among genes ATF3, ATF4, DDIT4, TRIB3 and FGD6 by the STRING interaction network. **B** Expression levels of ATF3, ATF4, DDIT4 and TRIB3 in HCT116, DLD-1 and HT-29 cells treated with DMSO or 100 nM sangivamycin for 24 h under monolayer conditions. Actin is the loading control. **C** Expression levels of ATF3, DDIT4 and TRIB3 were assessed in COAD tissues and normal adjacent tissues using RNA-Seq data from TCGA (* *p* < 0.05). Cohort size: (num)normal adjacent tissues = 349; (num) COAD tissues = 275. **D** Western blot analysis of the co-IP complex from cells treated with DMSO or 100 nM sangivamycin for 24 h with ATF3 antibody immobilized on Dynabeads®. Tubulin is the loading control for the input conditions. **E** Expression levels of ATF3, ATF4, DDIT4 and TRIB3 in HCT116 ATF3^parental^ and ATF3^KO^ cells treated with DMSO or 100 nM sangivamycin for 24 h under monolayer conditions. Actin is the loading control. **F** Illustration of the interaction between ATF3, ATF4, DDIT4 and TRIB3 based on our results and findings from other groups [79–81]. **G** ATF3 expression levels in HCT116 and HT-29 cells treated with the indicated concentrations of rapamycin for 72 h under monolayer conditions. Actin is the loading control. **H** ATF3 expression levels in HCT116 MCTS cells treated with rapamycin (10 µM) or Torin-1 (0.5 µM) for 72 h. Actin is the loading control. **I** Dose response of HCT116 and HT29 MCTS to 5-FU. The data is expressed as the mean ± SD (*n* = 3). The half-maximal inhibitory concentration (IC_50_) was calculated using nonlinear regression, [Inhibitor] vs. normalized response analysis. **J** Combination of rapamycin (10 µM) or Troin1(0.5 µM) with 5-FU under HCT116 MCTS conditions for 72 h. The data is expressed as the mean ± SD (*n* = 3, unpaired *t*-test, two-stage step-up (Benjamini, Krieger, and Yekutieli); * *p* < 0.05, *** *p* < 0.001). **K** Combination of rapamycin (10 µM) or Troin1(0.5 µM) with 5-FU under HT29 MCTS conditions for 72 h. CDI = AB/(A × B) where: AB = cell viability value for the combination of rapamycin/torin-1 and 5-FU. A and B = cell viability value for the single treatment rapamycin/torin-1 or 5-FU. A CDI value <1, =1 or >1 indicates that the drugs are synergistic, additive, or antagonistic, respectively. A CDI value less than 0.7 indicates a significant synergetic effect [31].

STRING interaction network further showed that at the protein level, TRIB3 and DDIT4 can interplay with the protein ATF3/4 (Fig. 6A, Supplementary Fig. S6A), and data from other groups revealed that TRIB3, DDIT4, ATF4 and ATF3 increased their expression levels under stressed conditions [59–61]. Thereby we hypothesized that ATF3/4, DDIT4 and TRIB3 may as well interrelate with each other at the protein level. To investigate this possibility, we first examined the expression levels of TRIB3, DDIT4, and ATF3/4, which did increase after 24 h incubation with sangivamycin (Fig. 6B). We also noted co-upregulation of TRIB3, DDIT4 and ATF3 expression levels between normal and tumor samples from TCGA COAD RNA-Seq data (Fig. 6C) and interrelated expression levels of TRIB3, DDIT4 and ATF3 in different cell types of colon tissue (Supplementary Fig. S6B, C), suggesting that DDIT4, TRIB3 and ATF3/4 could simultaneously increase in cells treated with sangivamycin. Second, we wondered in sangivamycin-treated samples whether the simultaneous increased expression of TRIB3, DDIT4, and ATF3/4 at the translational level was through direct interaction or indirect signal transduction. To answer this question, we performed co-IP assays by pulling down proteins associated with ATF3. It was interesting to note that ATF3 directly interacts with ATF4 but not TRIB3 and DDIT4 (Fig. 6D), leading us to speculate that the interaction of ATF3 with TRIB3 and DDIT4 is mainly mediated through indirect signal transduction. To understand this, we treated ATF3^parental^ and ATF3^KO^ cells with sangivamycin and checked the expression levels of TRIB3, DDIT4 and ATF3/4, respectively. In ATF3^KO^ cells, elevated levels of TRIB3, DDIT4, and ATF4 were still observed in sangivamycin-treated samples (Fig. 6E), indicating that ATF3 is a responder to DDIT4, TRIB3 and ATF4 (Fig. 6F).

Since ATF3 is a responder to DDIT4 and TRIB3, and could directly interact with ATF4, we speculated that any treatment that could suppress the expression level of DDIT4, TRIB3 or ATF4 may further decrease the ATF3 expression level. Rapamycin and torin-1 have been reported to decrease the expression level of DDIT4 and ATF4 [62–64]. We treated HCT116 and HT29 with the indicated concentrations of rapamycin or torin-1 and noticed a decrease in ATF3 after 72 h treatment under monolayer (Fig. 6G) and HCT116 MCTS conditions (Fig. 6H). Rapamycin showed negligible toxicity against HCT116 and HT29 MCTS up to 10 µM (Supplementary Fig. S6D). As high expression levels of ATF3 are a feature of QCCs that promotes resistance to anticancer reagents in CRC cells, we asked whether lowering ATF3 could improve the efficacy of drugs targeting the proliferating cancer cells. To test this possibility, we chose 5-FU and performed dose response on HCT116 and HT29 MCTS. 5-FU had an IC_50_ of 65 µM against HCT116 MCTS and 2790 µM against HT29 MCTS (Fig. 6I). We then combined indicated concentrations of 5-FU with rapamycin (10 µM) or torin-1(0.5 µM), and a synergistic effect was observed (Fig. 6J, K), suggesting that the combination of 5-FU with rapamycin or torin-1 can enhance the efficacy against CRC MCTS.

## Discussion

The lack of sufficient agents specifically killing the quiescent tumor cells that are insensitive to conventional cell-cycle targeted drugs is still a clinical challenge hampering the chemo-therapeutic effect. In this study, we performed single-cell RNA-Seq on the human colorectal carcinoma MCTS model [13, 33] and classified proliferating and quiescent cancer cell populations based on their specific markers [65, 66]. We then applied Gene Ontology pathway analysis and discovered significant changes in RNA polymerase II regulation, e.g., to stress and unfolded protein response in QCC populations, which is consistent with findings that imbalance of RNA polymerase II regulation provokes cell-cycle arrest [67] and unfolded protein response inhibits cell-cycle progression [68, 69]. Next, gene set enrichment analysis revealed the most significantly upregulated pathways in QCCs, including TNFα signaling via NF-kB, hypoxia and cholesterol homeostasis. Of note, the upregulation of pathways involved in cholesterol homeostasis in QCCs has been described in other studies [70, 71], suggesting that fatty acid metabolism could be interesting and thus is worthy of further investigation. Given that the top three pathways encapsulated the essential features of the two clusters (PCCs and QCCs), we posit that genes recurrent in these pathways hold considerable significance. ATF3 was one of three co-shared genes (ATF3, NFIL3, and PNRC1) and highly expressed in QCCs, suggesting its important role in the metabolically composed microenvironment. ATF3 (activating transcription factor 3) is a member of the ATF/cAMP response element-binding (CREB) family, which binds to the cyclic AMP response element (CRE) in numerous promoters with the consensus sequence TGACGTCA and acts as both a transcriptional activator and repressor [45, 72, 73]. Overwhelming evidence indicates that ATF3 plays an important role in metabolic regulation, immune responses, and oncogenesis. For example, ATF3 expression has been reported to be upregulated by low glucose and downregulated by high glucose [46], promote the serine synthesis pathway under dietary serine restriction [74], and regulate nucleotide metabolism [75]. Here we add another finding that elevated levels of ATF3 is a hallmark of QCCs. Since we also noticed that the expression of ATF3 gradually decreased along the pseudotime PCC trajectory, this led us to speculate that the expression level of ATF3 could also play an essential role in cell-cycle regulation, and compounds upregulating ATF3 expression, such as sangivamycin, could prevent repopulation of quiescent cancer cells, encouraging us to study the possibility of using sangivamycin between chemotherapy cycles to delay tumor recurrence. Besides ATF3, NFIL3 (nuclear factor, interleukin 3) and PNRC1 (proline-rich nuclear receptor coactivator 1) were the other two co-shared genes of the top 3 altered signaling pathways in QCCs, which deserve further investigation, especially PNRC1, whose ablation has been reported to enhance the effects of RAS and MYC [76].

In addition, we carried out a chemical screening using the mechanistic set provided by the National Institutes of Health (NCI) and discovered three hit compounds that exhibited specific toxicity against ATF3-deficient cells. NSC 328587 was reported to inhibit glycolysis [77], NSC 177365 could repress hTERT transcription, and NSC359463 inhibits Cathepsin K [78]. However, we did not identify any compounds targeting cells expressing ATF3. Combined with the finding that cells with the ability to upregulate ATF3 exhibited greater resistance to sangivamycin, we conclude that ATF3 has a protective role in metabolically compromised microenvironments.

Currently, the mechanisms by which ATF3 regulates transcription remain largely unknown. Here, we found that ATF3 can interact with both DDIT4 and TRIB3 at the transcriptional level and with ATF4 at the translational level. Furthermore, DDIT4, TRIB3 and ATF3 were co-upregulated in CRC patient samples, suggesting that regulation between ATF3, DDIT4, and TRIB3 is clinically important. Since ATF3 is highly expressed in QCCs, further study of this finding may help us better understand the behavior of QCCs, and certain treatments that impair ATF3-enhanced cytoprotection may improve the outcome of CRC treatment, such as the combination of 5-FU with rapamycin or torin-1 investigated in this study.

One of the potentially important questions that we were not able to address in this paper is the identity of Group 3 discovered by scRNA-Seq with high expression levels of quiescent and proliferative markers. Our additional data showed that group 3 starts at the earliest pseudotime, where groups 1, 2 and 3 co-exist (Supplementary Fig. S3B). The gene ontology terms analysis illuminated that Group 3 was enriched in biological functions pertaining to cytoplasmic translation, maintenance of location in cell and regulation of generation of precursor metabolites and energy pathway (Supplementary Fig. S3C). Interestingly, IHC staining of HCT116 MCTS found some cells in the core (p27 high) are as well with high Ki67 expression levels, which may belong to the undefined group 3, encouraging us to emphasize this point in future studies. It is also worth mentioning that QCCs and PCCs in MCTS can be collected based on specific markers (such as p27 and Ki67) using flow cytometric analysis.

In conclusion, in this study, we identified that elevated levels of ATF3 is a hallmark of QCCs, and novel therapeutic combinations that could lower the expression level of ATF3 warrant further investigation.

**Key resources table**

**Table:** 

Product name	Catalog number	Company
*Chemicals*		
Sangivamycin	HY-118384	MedChemExpress
Rapamycin	HY-10219	MedChemExpress
Torin-1	HY-13003	MedChemExpress
*Antibodies*		
Rabbit anti-ATF3 (WB)	#33593	Cell Signaling
Rabbit anti-ATF4	#118115	Cell Signaling
Rabbit anti-TRIB3	#43043	Cell Signaling
Rabbit anti-DDIT4	#10638-1-AP	Proteintech
Mouse anti-actin	#sc-47778	Santa Cruz Biotechnology
Mouse anti-tubulin	#sc-5286	Santa Cruz Biotechnology
Rabbit anti-ATF3 (IP)	#18665	Cell Signaling
Secondary anti-Rabbit	#31430	Thermo Scientific
Secondary anti-Mouse	#31460	Thermo Scientific
Ki67(IHC)	#M7240	Agilent Technologies
p27	#M7203	Agilent Technologies
ATF3	#MA5-31360	Thermo Scientific
Anti-Mouse IgG Reagent	#MP-7402	Vector Laboratories
DAB Substrate Kit	#SK-4100	Vector Laboratories
*Cell lines*		
HCT116	#CCL-247	ATCC
DLD-1	#CCL-221	ATCC
HT-29	#HTB-38	ATCC
HEK293T	#CRL-3216	ATCC
*Reagents and consumables*		
McCoy’s 5A	#16600082	Thermo Scientific
DMEM medium	#10569010	Thermo Scientific
Fetal Bovine Serum	#10270106	Thermo Scientific
Penicillin-Streptomycin	#15140-122	Thermo Scientific
Resazurin powder	#R7017	Sigma-Aldrich
Antigen unmasking solution	#H-3300	Vector Laboratories
2.5% Normal Horse Serum	#S-2012	Vector Laboratories
Hematoxylin	#S3309	Agilent Technologies
Mounting buffer	#S3025	Agilent Technologies
Tris-Cl PH 8.0	#15568-025	Invitrogen
EDTA	#AM9290G	Invitrogen
1% SDS	#15553-035	Invitrogen
0.2 M NaCl	#AM9759	Invitrogen
Proteinase K	#EO0491	Thermo Scientific
Histogel	#HG-4000-012	Fisher Scientific
96-well ultra-low attachment plates	#7007	Corning
RIPA lysis buffer	#89901	Fisher Scientific
4–12% SDS-PAGE	#NP0336BOX	Thermo Scientific
MinElute PCR Purification kit	#28004	QIAGEN
Dynabeads™ Co-Immunoprecipitation Kit	#14321D	Thermo Scientific

### Supplementary information

Supplementary Information file

Original Data File

aj-checklist

Table S1.xls

Table S2.xls

Table S3.xls

TableS4.xls

Table S5.xls

Table S6.xls

Table S7.xls

Table S8.xls

## Data Availability

The sequencing data generated were deposited in the Short Read Archive under project number PRJNA1013370. All detailed scripts used in this study were deposited and can be accessed via https://github.com/Summertree23/ATF3 (https://zenodo.org/record/8320183). All original western blots can be found in the Supplementary Material-uncropped western blot.
