# Supplementary material for: Identification of ATF3 as a novel protective signature of quiescent colorectal tumor cells
Source: Cell Death Dis. 2023 Oct 13;14(10):676. doi: 10.1038/s41419-023-06204-1 (PMC10576032; doi:10.1038/s41419-023-06204-1)
Supplement: Supplementary file 1 — Supplementary Information file [file 41419_2023_6204_MOESM1_ESM.pdf]

Supplementary method

Human Protein Atlas

In order to provide additional background information, expression data for ATF3, DDIT4, and TRIB3 was sourced from the Human Protein Atlas (HPA) from its respective entry page for each gene in the proposed regulatory axis.[1] The RNA single cell subsection on HPA served as the source for representative RNA expression data in normal colon tissue at single cell resolution. Additionally, the HPA provided a heatmap of normalized expression values of the single cell expression data, which were further modified for each gene in order to facilitate comparison across genes in the proposed regulatory axis as well comparison of the genes against expression of known canonical cell-type markers for normal colon tissue.[2,3,4]

References

1 Karlsson M et al., A single-cell type transcriptomics map of human tissues. Sci Adv. (2021) PubMed: 34321199 DOI: 10.1126/sciadv.abh2169

2 Image source: The Human Protein Atlas, "Single Cell Type Colon: ATF3" 2023. Accessed via https://www.v22.proteinatlas.org/ENSG00000162772-ATF3/single+cell+type/colon

3 Image source: The Human Protein Atlas, "Single Cell Type Colon: DDIT4" 2023. Accessed via https://www.v22.proteinatlas.org/ENSG00000168209-DDIT4/single+cell+type/colon

4 Image source: The Human Protein Atlas, "Single Cell Type Colon: TRIB3" 2023. Accessed via https://www.v22.proteinatlas.org/ENSG00000101255-TRIB3/single+cell+type/colon

Supplementary Figure Legend

Figure S1

A. The correlation between number of counts/cell and the number of detected genes/cell.
B. The percent of mitochondria per cell.

Figure S2

A. UMAP of indicated genes, ATF3, ATF4, PNRC1 and NFL3.
B. Images of HCT116, HT-29, DLD-1 and HEK293T MCTS models, taken by Incucyte Scale bar: 500µm.
C. UMAP plots showing in total eight clusters. Group 0 and Group 1 are immune cell subsets, Group 2,3 5 6 and 7 are tumor cells subsets. Group 4 is CD45-/ vimentin-.
D. expression levels of cell cycle active, cell cycle inactive, QCC-dependent marker ATF3 and indicated proteins discovered from ATAC-Seq.

Figure S3

A. Percentage of spliced and unspliced mRNA in PCCs and QCCs.
B. A heatmap representation of the pseudotime inference three groups.
C. Gene Ontology terms are represented based on genes that correlated with pseudotime for Group 3.
D. The dynamical model of indicated genes (MKI67, CDK4, CDKN1A, CDKN1C and ATF3) suggest that MKI67 and CDK4 have high likelihoods to associate with PCCs, CDKN1A, CDKN1C and ATF3 have high likelihoods to associate with QCCs.

Figure S4

A. Growth rate of ATF3 parental and ATF3 KO HCT116 clones in monolayer and MCTS conditions. The data is expressed as the mean ± SD (n=3, using multiple-unpaired t-test).
B. Expression levels of ATF3 in scramble and siATF3 HT-29 cells were detected after 48hs transfection.
C. Chemical structure of sangivamycin (4-amino-5-carboxamide-7-(d-ribofuranosyl)pyrrolo [2,3-d] pyrimidine).

Figure S5

A. Pearson correlation of read counts comparing between two untreated samples and two treated samples.
B. Heatmap showing correlations and hierarchical clustering for read densities at open chromatin peaks across all ATAC-Seq replicates.
C. Genome browser tracks of ATAC-Seq data for FGD6.

Figure S6
A. Plot for protein interactions among genes annotated to the chromatin regions in samples treated with sangivamycin by the STRING interaction network.
B. Overview of transcriptomic level gene expression for ATF3, DDIT4 and TRIB3 at single cell resolution in normal colon tissue.
C. Heatmap comparing the expression of the selected genes to established canonical cell-type markers for colon tissue. Expression data for ATF3, DDIT4 and TRIB3 from the Human Protein Atlas (HPA).
D. Dose response of HCT116 and HT29 MCTS to rapamycin.

Fig. S1

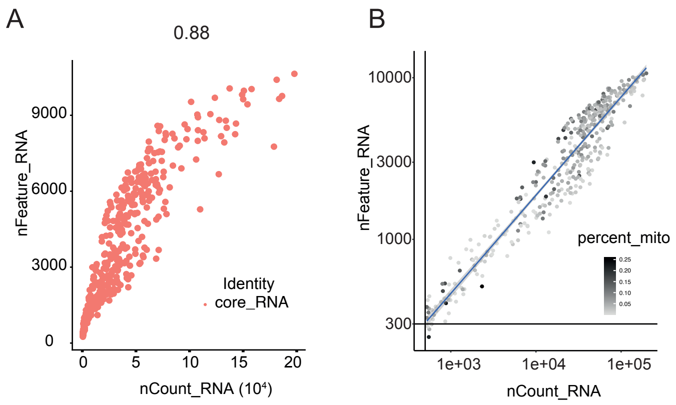

Figure S1

A. The correlation between number of counts per cell and the number of detected genes per cell.

B. The percent of mitochondria per cell.

Fig. S2

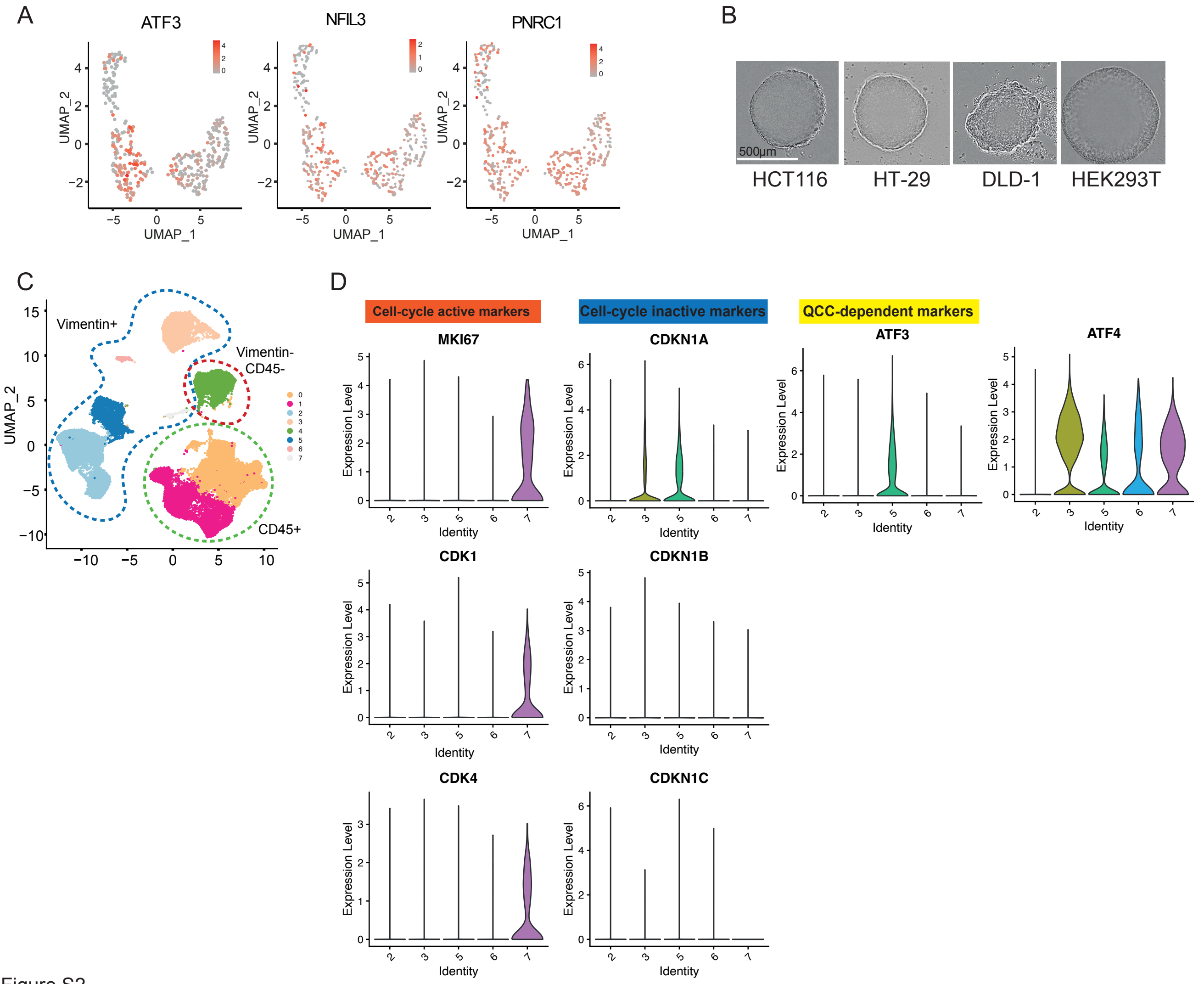

Figure S2

A. UMAP of indicated genes, ATF3, PNRC1 and NFL3.

B. Images of HCT116, HT-29, DLD-1 and HEK293T MCTS models, taken by Incucyte Scale bar: 500μm.

C. UMAP plots showing in total eight clusters. Group 0 and Group 1 are immune cell subsets, Group 2,3 5 6 and 7 are tumor cells subsets. Group 4 is CD45-/ vimentin-.

D. Expression levels of cell cycle active, cell cycle inactive, QCC-dependent marker ATF3 and ATF4.

Fig. S3

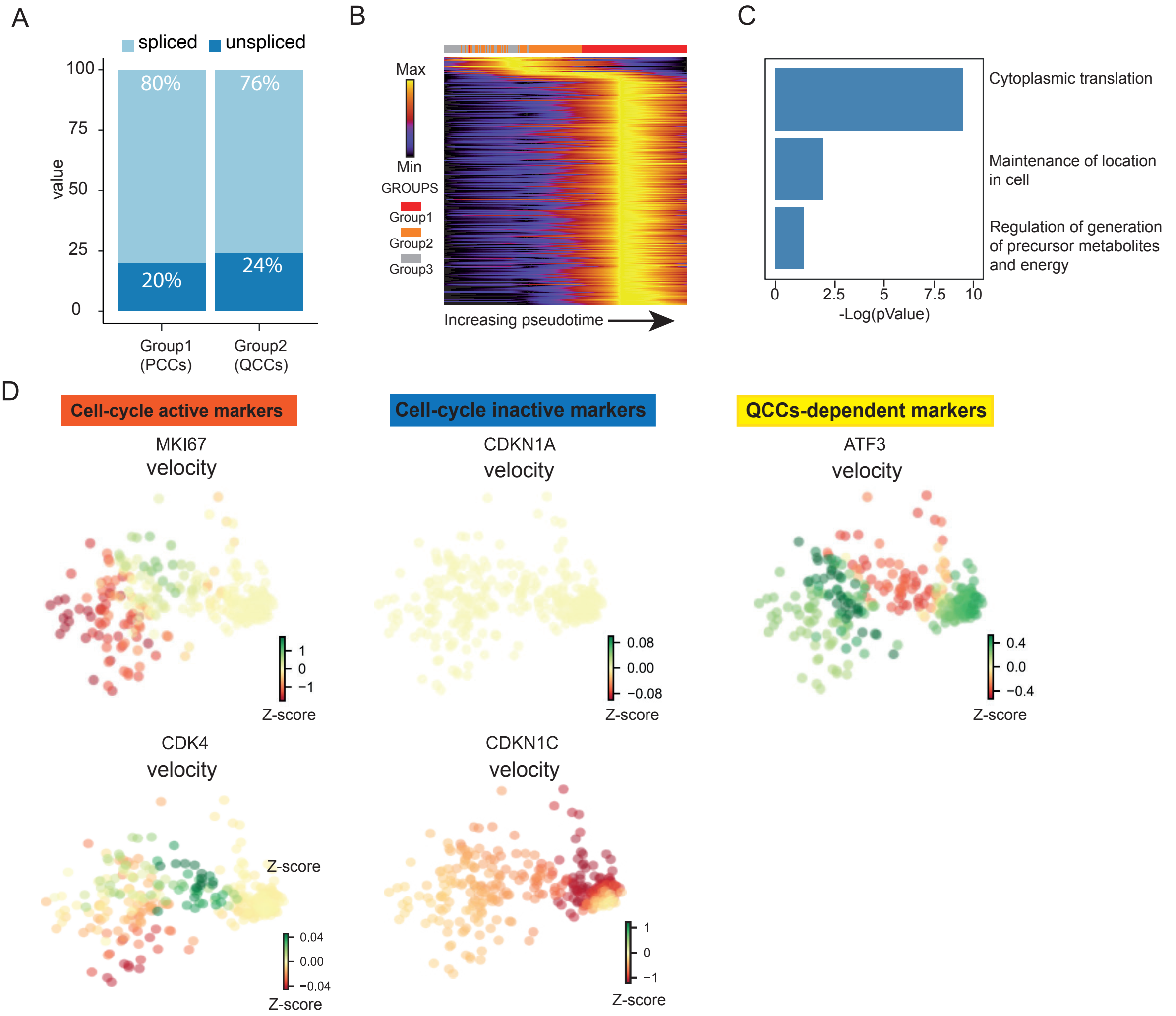

Figure S3
A. Percentage of spliced and unspliced mRNA in PCCs and QCCs.
B. A heatmap representation of the pseudotime inference three groups.
C. Gene Ontology terms are represented based on genes that correlated with pseudotime for Group 3.
D. The dynamical model of indicated genes (MKI67, CDK4, CDKN1A, CDKN1C and ATF3) suggest that MKI67 and CDK4 have high likelihoods to associate with PCCs; CDKN1A, CDKN1C and ATF3 have high likelihoods to associate with QCCs.

Fig. S4

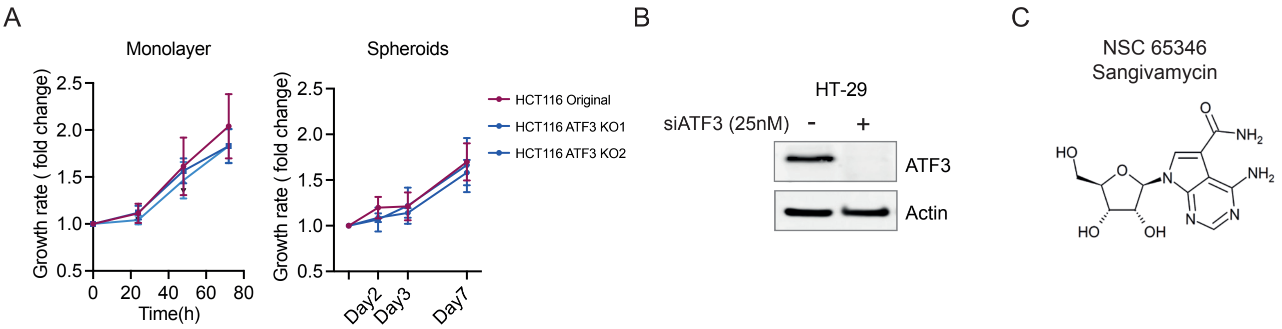

Figure S4

A. Growth rate of ATF3^{parental} and ATF3^{KO} HCT116 clones in monolayer and MCTS conditions. The data is expressed as the mean ± SD (n=3, using multiple-unpaired t-test).

B. Expression levels of ATF3 in scramble and siATF3 HT-29 cells were detected after 48hs transfection.

C. Chemical structure of sangivamycin (4-amino-5-carboxamide-7-(d-ribofuranosyl)pyrrolo [2,3-d] pyrimidine).

Fig. S5

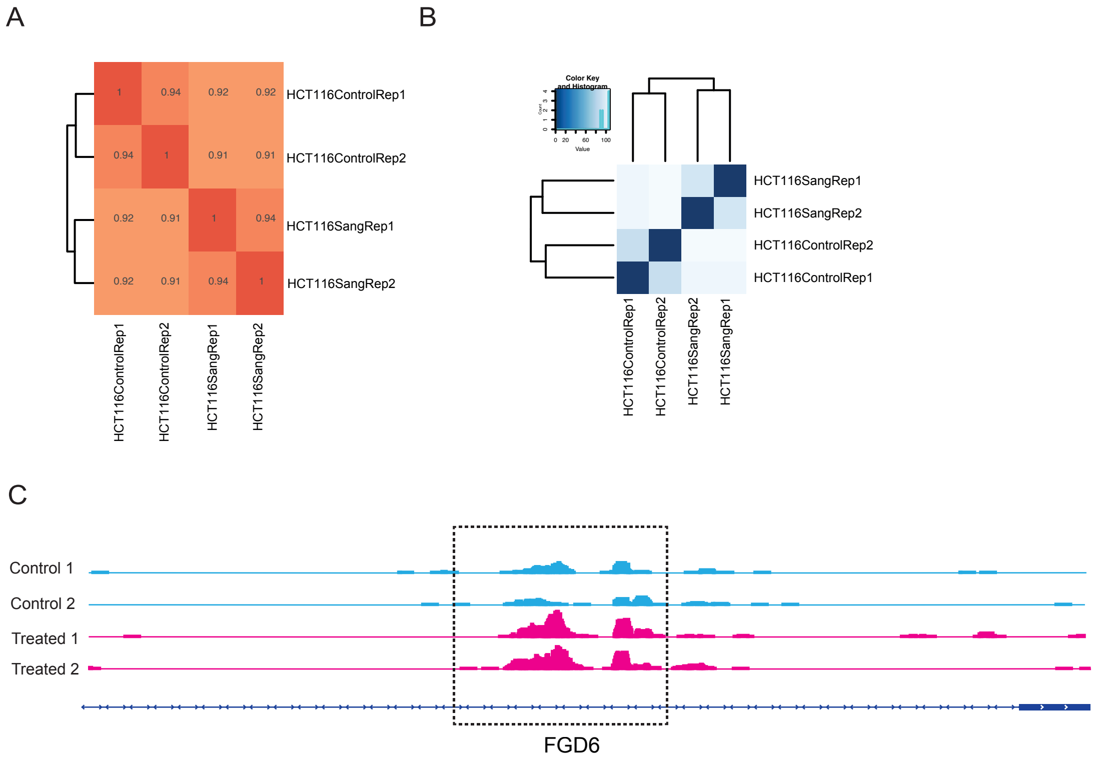

Figure S5

A. Pearson correlation of read counts comparing between two untreated samples and two treated samples.

B. Heatmap showing correlations and hierarchical clustering for read densities at open chromatin peaks across all ATAC-Seq replicates.

C. Genome browser tracks of ATAC-Seq data for FGD6.

Fig. S6

A

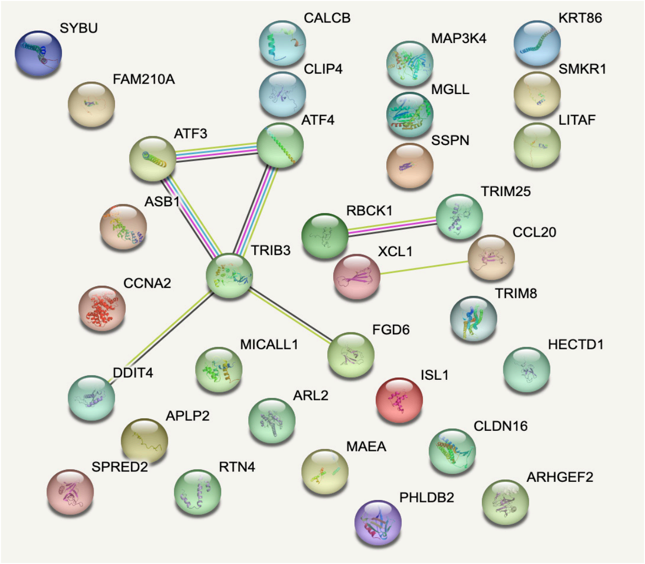

D

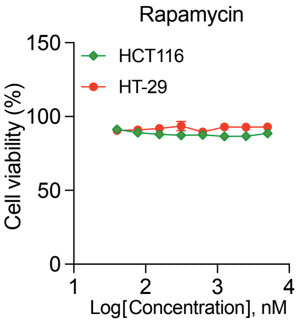

Figure S6

A. Plot for protein interactions among genes annotated to the chromatin regions in samples treated with sangivamycin by the STRING interaction network.

B. Overview of transcriptomic level gene expression for ATF3, DDIT4 and TRIB3 at single cell resolution in normal colon tissue.

C. Heatmap comparing the expression of the selected genes to established canonical cell-type markers for colon tissue. Expression data for ATF3, DDIT4 and TRIB3 from the Human Protein Atlas (HPA).

D. Dose response of HCT116 and HT29 MCTS to rapamycin.

Fig. S6

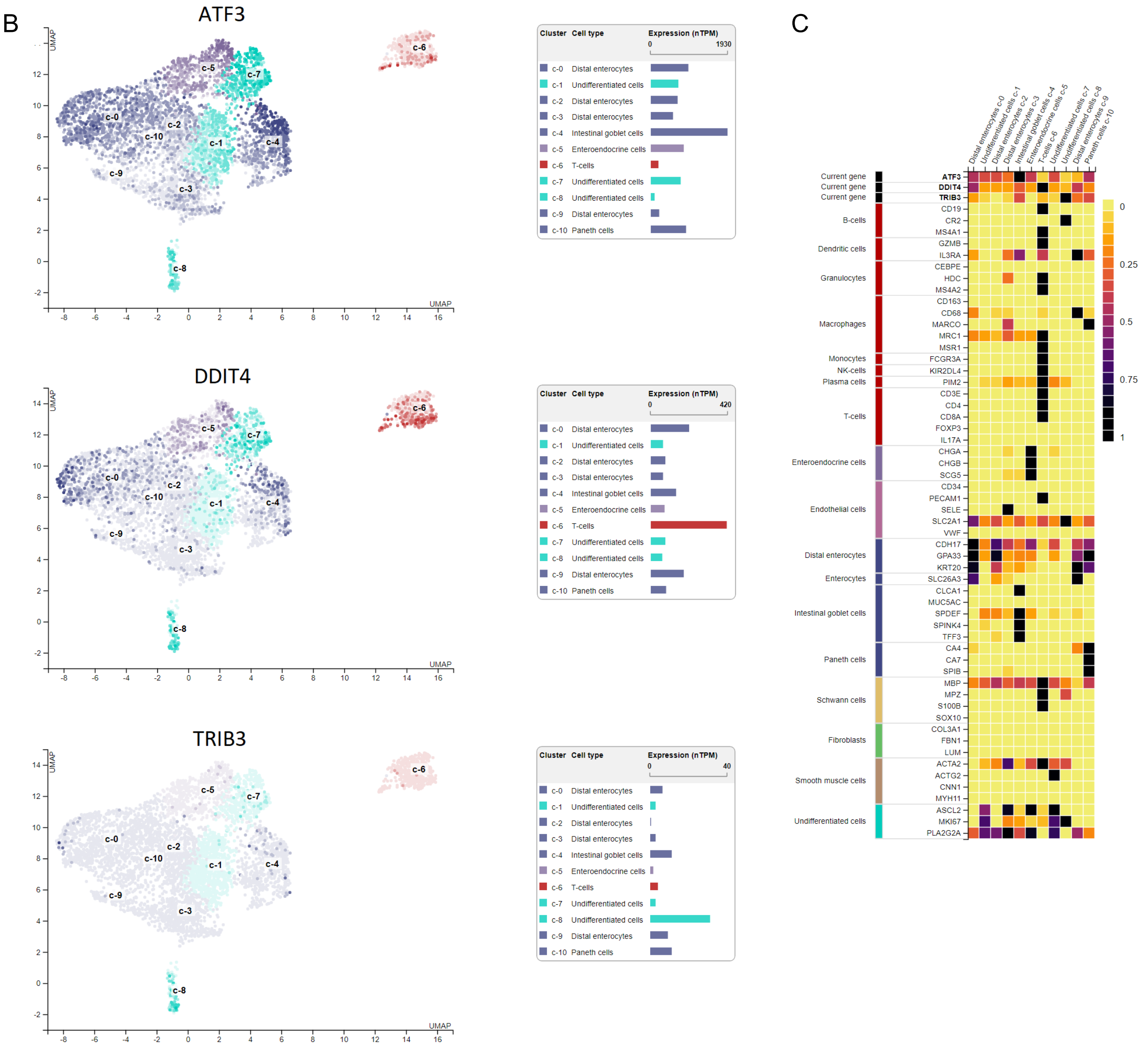

B
ATF3
DDIT4
TRIB3
UMAP
c-0
c-1
c-2
c-3
c-4
c-5
c-6
c-7
c-8
c-9
c-10
Cluster
Cell type
Expression (nTPM)
0
1930
420
40
Distal enterocytes
Undifferentiated cells
Intestinal goblet cells
Enteroendocrine cells
T-cells
Paneth cells
C
Distal enterocytes c-0
Undifferentiated cells c-1
Distal enterocytes c-2
Distal enterocytes c-3
Intestinal goblet cells c-4
Enteroendocrine cells c-5
T-cells c-6
Undifferentiated cells c-7
Undifferentiated cells c-8
Distal enterocytes c-9
Paneth cells c-10
Current gene
ATF3
DDIT4
TRIB3
B-cells
CD19
CR2
MS4A1
Dendritic cells
GZMB
IL3RA
Granulocytes
CEBPE
HDC
MS4A2
Macrophages
CD163
CD68
MARCO
MRC1
MSR1
Monocytes
FCGR3A
NK-cells
KIR2DL4
Plasma cells
PIM2
T-cells
CD3E
CD4
CD8A
FOXP3
IL17A
Enteroendocrine cells
CHGA
CHGB
SCG5
Endothelial cells
CD34
PECAM1
SELE
SLC2A1
VWF
Distal enterocytes
CDH17
GPA33
KRT20
Enterocytes
SLC26A3
Intestinal goblet cells
CLCA1
MUC5AC
SPDEF
SPINK4
TFF3
Paneth cells
CA4
CA7
SPIB
Schwann cells
MBP
MPZ
S100B
SOX10
Fibroblasts
COL3A1
FBN1
LUM
Smooth muscle cells
ACTA2
ACTG2
CNN1
MYH11
Undifferentiated cells
ASCL2
MKI67
PLA2G2A
0
0.25
0.5
0.75
1
