# Supplementary material for: Identification of ATF3 as a novel protective signature of quiescent colorectal tumor cells
Source: Cell Death Dis. 2023 Oct 13;14(10):676. doi: 10.1038/s41419-023-06204-1 (PMC10576032; doi:10.1038/s41419-023-06204-1)

Figure 2G

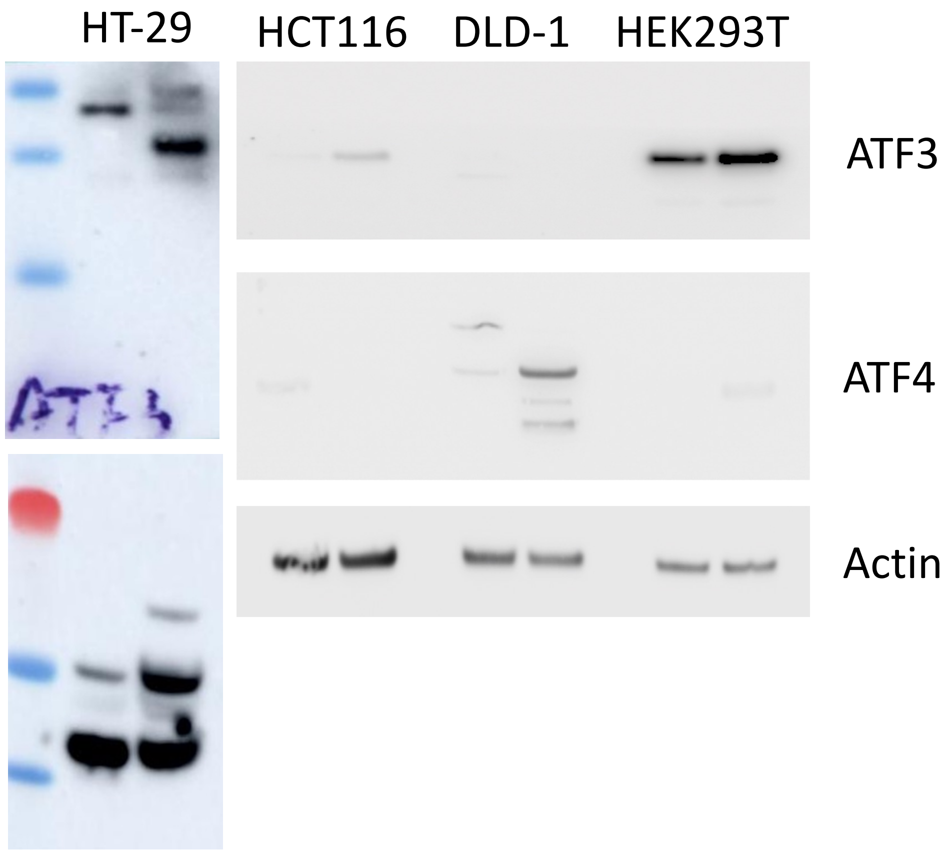

Figure 4A

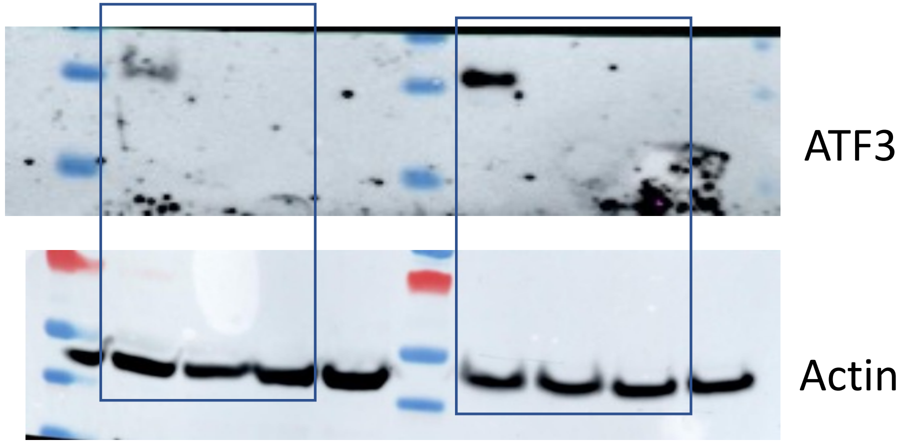

Figure 4I

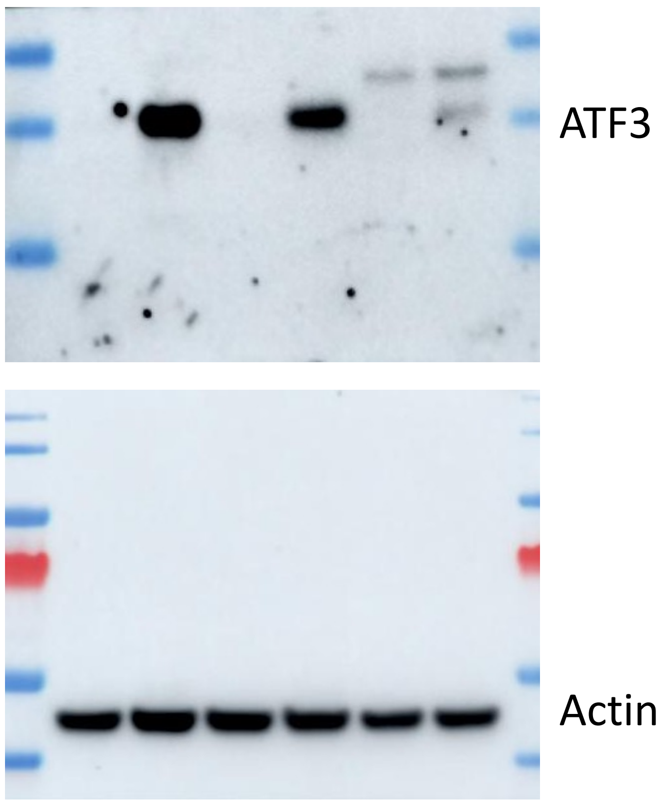

Figure 4J

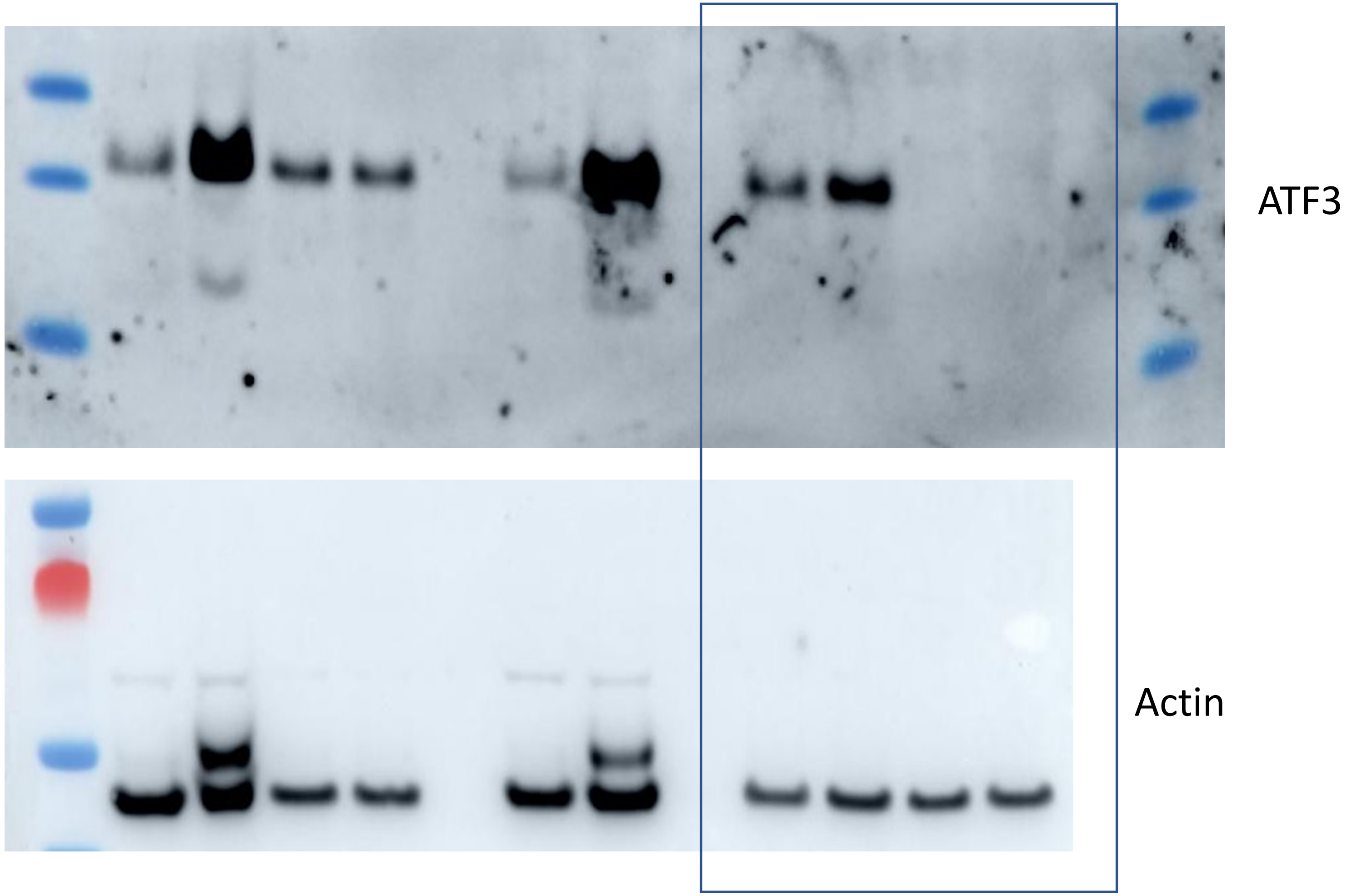

Figure 6B

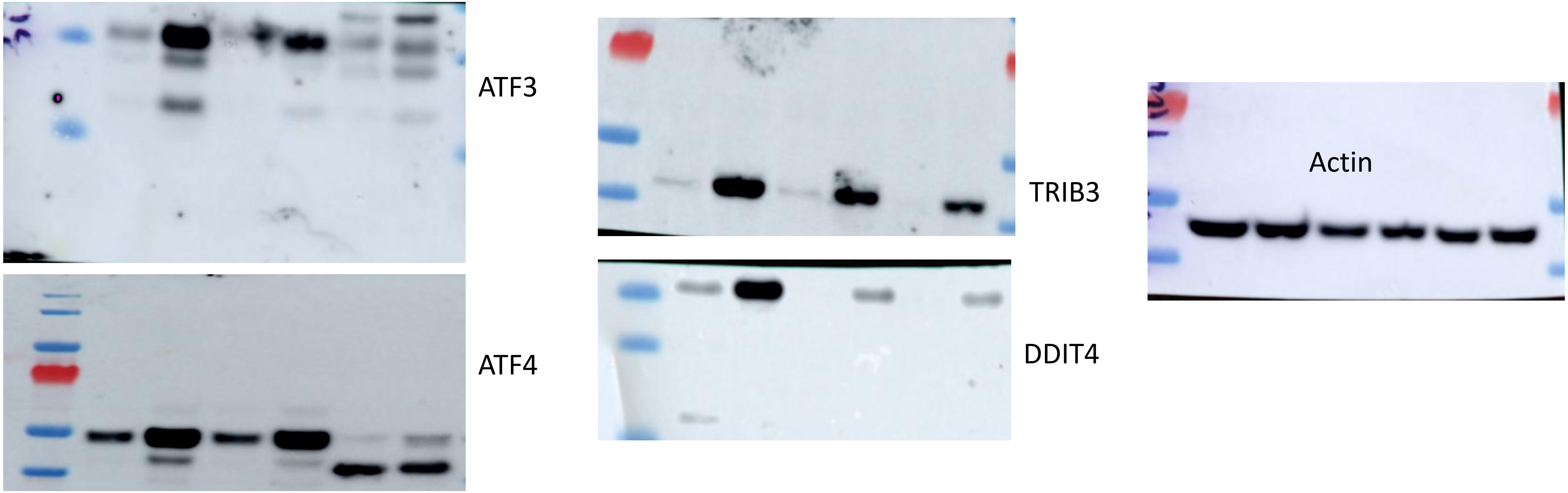

Figure 6D

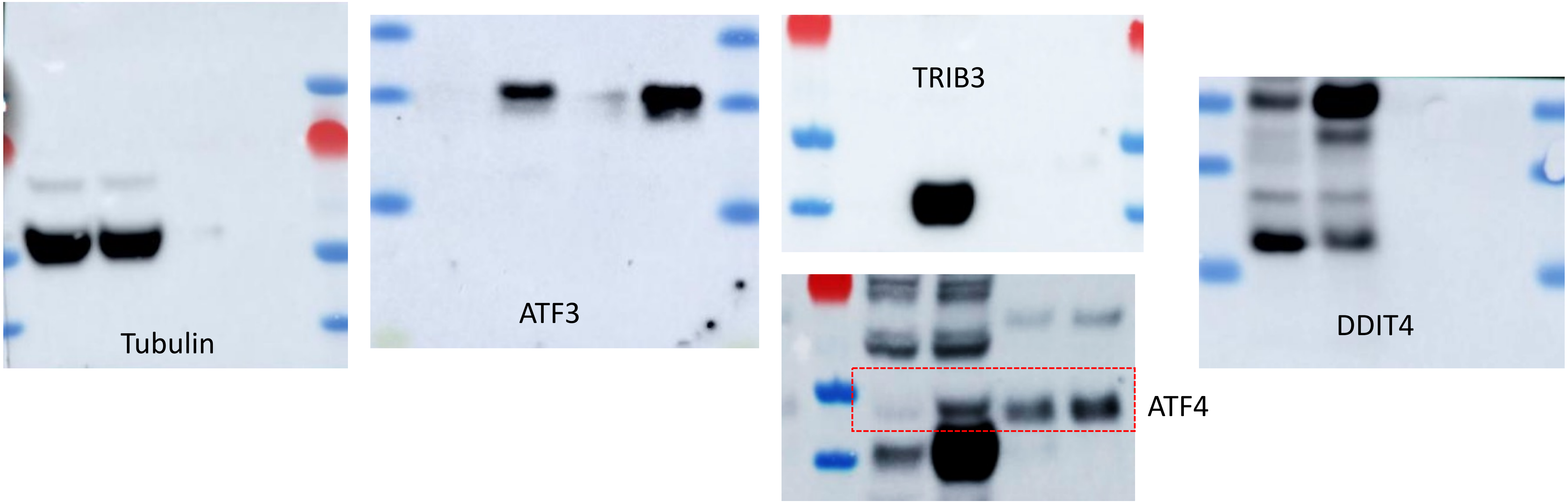

Figure 6E

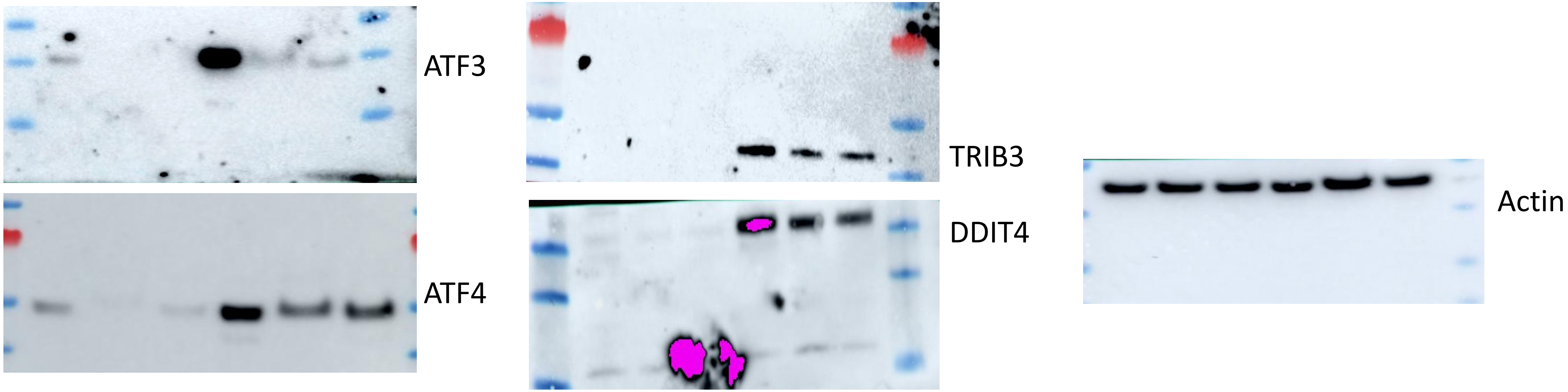

Figure 6G

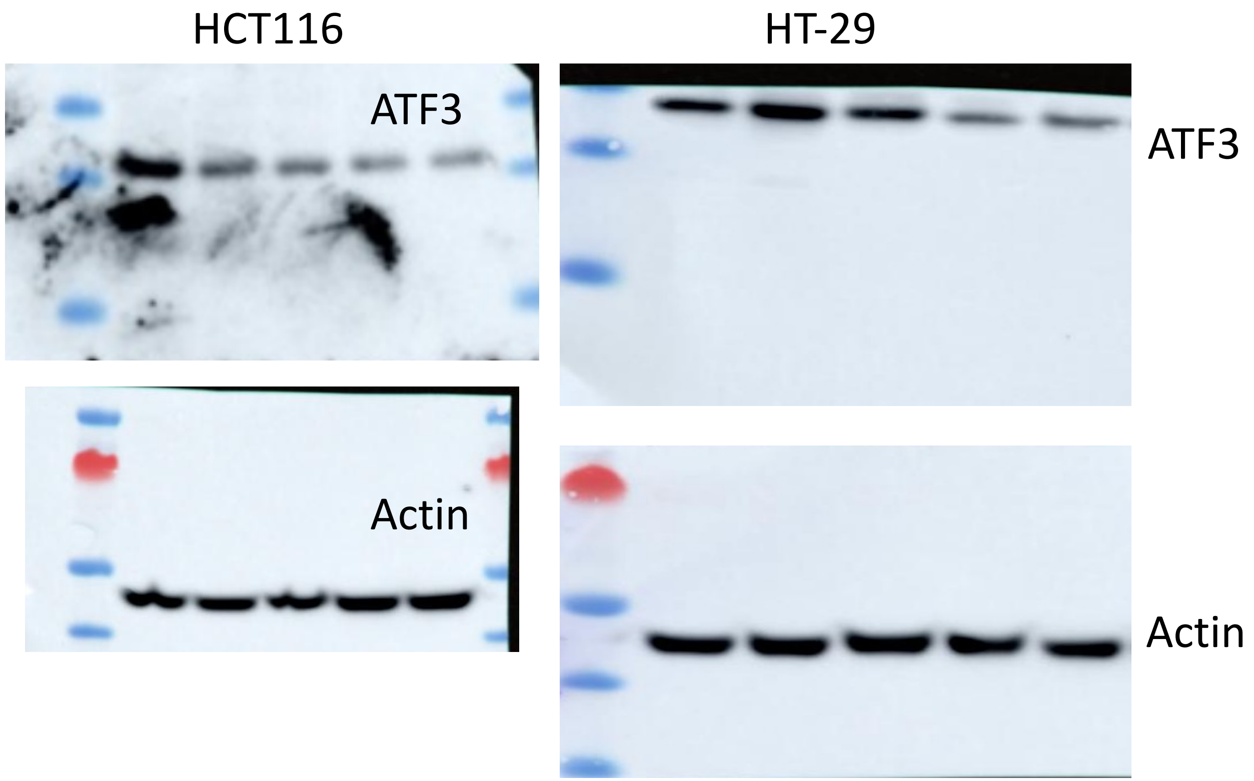

Figure 6H

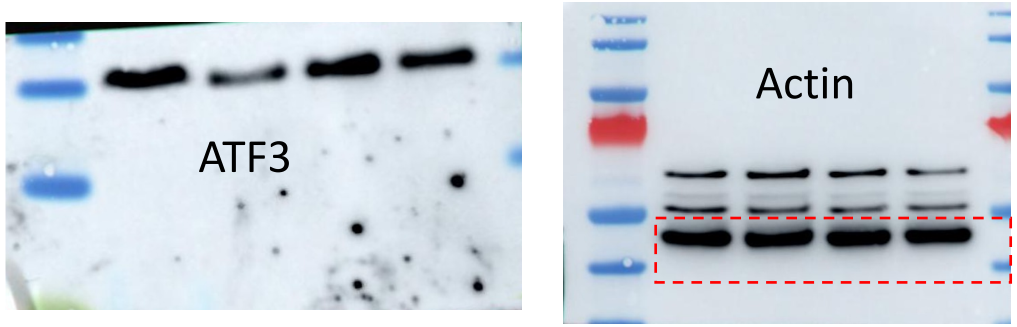

Fig. S4B

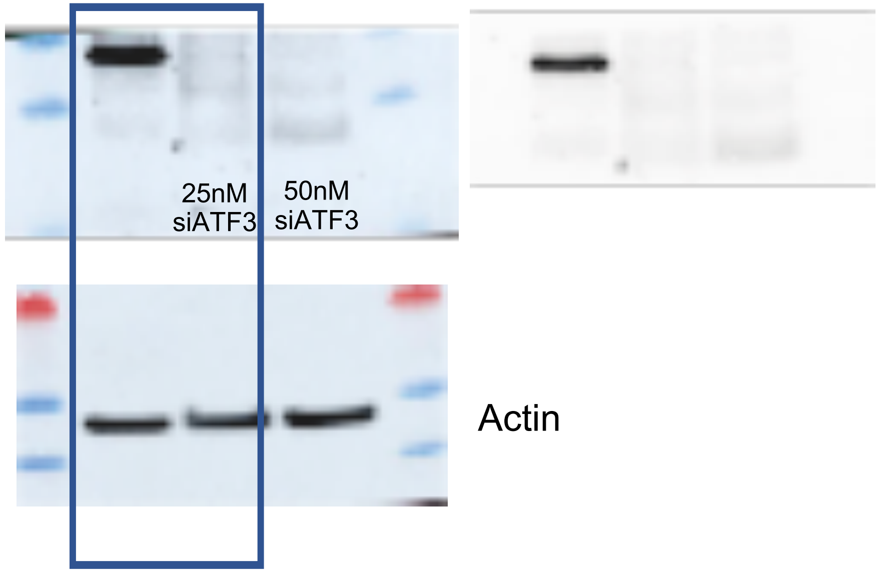

Supplement: Supplementary file 2 — Original Data File [file 41419_2023_6204_MOESM2_ESM.pdf]
